# Crystal structure and interaction studies of human DHTKD1 provide insight into a mitochondrial megacomplex in lysine catabolism

**DOI:** 10.1107/S205225252000696X

**Published:** 2020-06-10

**Authors:** Gustavo A. Bezerra, William R. Foster, Henry J. Bailey, Kevin G. Hicks, Sven W. Sauer, Bianca Dimitrov, Thomas J. McCorvie, Jürgen G. Okun, Jared Rutter, Stefan Kölker, Wyatt W. Yue

**Affiliations:** a Structural Genomics Consortium, Nuffield Department of Medicine, University of Oxford, Oxford, OX3 7DQ, United Kingdom; b Department of Biochemistry, University of Utah School of Medicine, USA; c Division of Child Neurology and Metabolic Medicine, Centre for Pediatrics and Adolescent Medicine, Clinic I, University Hospital Heidelberg, Germany

**Keywords:** human DHTKD1, 2-oxoadipate, 2-oxoacid de­hydrogenase, thi­amine diphosphate, lysine catabolism, cryo-EM, enzyme mechanisms, multi-protein complexes

## Abstract

DHTKD1 is a lesser-studied E1 enzyme among the family of 2-oxoacid de­hydrogenases. In complex with E2 (di­hydro­lipo­amide succinyltransferase, DLST) and E3 (dihydrolipo­amide de­hydrogenase, DLD) components, DHTKD1 is involved in lysine and tryptophan catabolism by catalysing the oxidative de­carboxyl­ation of 2-oxoadipate (2OA) in mitochondria. Here, the 1.9 Å resolution crystal structure of human DHTKD1 is solved in complex with the thi­amine diphosphate co-factor. The structure reveals how the DHTKD1 active site is modelled upon the well characterized homologue 2-oxoglutarate (2OG) de­hydrogenase but engineered specifically to accommodate its preference for the longer substrate of 2OA over 2OG. A 4.7 Å resolution reconstruction of the human DLST catalytic core is also generated by single-particle electron microscopy, revealing a 24-mer cubic scaffold for assembling DHTKD1 and DLD protomers into a megacomplex. It is further demonstrated that missense DHTKD1 variants causing the inborn error of 2-amino­adipic and 2-oxoadipic aciduria impact on the complex formation, either directly by disrupting the interaction with DLST, or indirectly through destabilizing the DHTKD1 protein. This study provides the starting framework for developing DHTKD1 modulators to probe the intricate mitochondrial energy metabolism.

## Abstract 2

Through interaction studies, the crystal structure of human DHTKD1 allows insight into a mitochondrial megacomplex in lysine catabolism. This creates the starting framework for developing DHTKD1 modulators to probe the intricate mitochondrial energy metabolism.

## 1. Introduction   

The family of multi-component 2-oxoacid de­hydrogenase complexes, of which pyruvate de­hydrogenase (PDHc), branched chain α-ketoacid de­hydrogenase (BCKDHc) and 2-oxoglutarate de­hydrogenase (OGDHc) complexes are canonical members, catalyse the oxidative de­carboxyl­ation of 2-oxoacids (*e.g.* pyruvate, 2-oxoisovalerate and 2-oxoglutar­ate) into their corresponding acyl-CoA thio­esters, generating the reducing equivalent NADH (nicotinamide adenine di­nucleotide in a reduced form). These biochemical reactions play crucial roles in intermediary metabolism, and are tightly regulated by phospho­rylation and allosteric effectors (Yeaman, 1989 ▸; Reed, 2001 ▸).

The overall reaction catalysed by 2-oxoacid de­hydrogenases is dissected into three sequential steps each catalysed by an individual enzyme (Perham, 1991 ▸; Jordan, 2003 ▸). In the first step, rate limiting for the overall reaction, the E1 enzyme (a 2-oxoacid de­carboxyl­ase; EC 1.2.4.2) catalyses the irreversible de­carboxyl­ation of 2-oxoacids via the thi­amine diphosphate (ThDP) co-factor and subsequent transfer of the de­carboxyl­ated acyl intermediate on an oxidized di­hydro­lipoyl group that is covalently amidated to the E2 enzyme (a di­hydro­lipoyl acyl­transferase; EC 2.3.1.61). In the second step, E2 transfers the acyl moiety from the di­hydro­lipoyl group onto a CoA-SH acceptor, generating acyl-CoA and a reduced di­hydro­lipoyl group. In the final step, one FAD-dependent (flavin adenine dinucleotide) E3 enzyme universal to all complexes (di­hydro­lipo­amide de­hydrogenase, DLD; EC 1.8.1.4) re-oxidizes the di­hydro­lipoyl group by transferring one reducing equivalent of NAD^+^ to yield NADH.

To achieve the overall oxidative de­carboxyl­ation reaction, multiple copies of the E1, E2 and E3 components classically assemble into a supramolecular complex reaching 4–10 MDa in weight (Marrott *et al.*, 2014 ▸). Structural studies have shown E2 enzymes from various organisms to exist in a high-order cubic 24-mer or dodecahedral 60-mer (Izard *et al.*, 1999 ▸), acting as a scaffold onto which copies of E1 and E3 are assembled. Such a quaternary arrangement, a classic example of a metabolon, provides a means by which products of one reaction are funnelled into the catalytic centres of the next reactions to enhance enzymatic efficiency and avoid undesirable side reactions (Cohen & Pielak, 2017 ▸). For example, the E2-attached di­hydro­lipoyl co-factor is expected to shuttle catalytic intermediate substrates between E1 and E3 enzymes by means of a ‘swinging-arm’ mechanism (Zhou *et al.*, 2001 ▸; Reed & Hackert, 1990 ▸; Perham *et al.*, 2002 ▸).

The human genome encodes five E1-type de­carboxyl­ases (PDH, BCKDH, OGDH, DHTKD1 and OGDHL), among which OGDH, OGDHL and DHTKD1 form a more evolutionarily related subgroup with respect to the E1 architecture and the E2 enzyme employed (Bunik & Degtyarev, 2008 ▸). The OGDHc complex, composed of OGDH as E1, di­hydro­lipo­amide succinyltransferase (DLST) as E2 and DLD as E3, converts the metabolite 2-oxoglutarate (2OG) to succinyl-CoA and serves as a rate-limiting step in the Krebs cycle (Araújo *et al.*, 2013 ▸). A close homologue of OGDH, the OGDH-like protein (OGDHL) is expressed in the brain (Bunik *et al.*, 2008 ▸) and implicated in brain pathways of glutamate and Ca^2+^ sensing. While its precise physiological role is not defined, OGDHL is considered as a tissue-specific isoenzyme of OGDH. A second analogue of OGDH, the enzyme DHTKD1 (de­hydrogenase E1 and transketolase domain-containing protein 1) is positioned in the last step of lysine and tryptophan catabolism with the common product being 2-oxoadipate (2OA), one methyl­ene group longer than 2OG. To catalyse the oxidative de­carboxyl­ation of 2OA to glutaryl-CoA (Nemeria, Gerfen, Yang *et al.*, 2018 ▸), DHTKD1 recruits the same E2 (DLST) and E3 (DLD) as OGDH to form the 2-oxoadipate de­hydrogenase complex (OADHc) (Goncalves *et al.*, 2016 ▸; Nemeria, Gerfen, Nareddy *et al.*, 2018 ▸), implying that DLST also acts as a di­hydro­lipo­amide glutaryl­transferase. Both DHTKD1 (Quinlan *et al.*, 2014 ▸; Bunik & Brand, 2018 ▸) and OGDH (Xu *et al.*, 2013 ▸; Sherrill *et al.*, 2018 ▸) are emerging as contributors of reactive oxygen species in mitochondria, through catalysing a side reaction in the forward reaction that results in superoxide/H_2_O_2_ formation (Goncalves *et al.*, 2016 ▸; Bunik & Sievers, 2002 ▸). The identification of DHTKD1 as an additional reactive oxygen species (ROS) source in mitochondria implies a contribution to oxidative stress under pathophysiological conditions such as those associated with mitochondrial abnormalities and neurodegeneration (Jordan *et al.*, 2019 ▸). To this end, DHTKD1 is increasingly recognized as essential for mitochondrial function and energy production, whereby loss of DHTKD1 function is associated with decreased adenosine triphosphate (ATP) production, increased ROS production and impaired mitochondrial biogenesis in cultured cells (Xu *et al.*, 2013 ▸; Sherrill *et al.*, 2018 ▸).

In support of this role, inherited DHTKD1 mutations are identified as the molecular cause of two rare Mendelian disorders. 2-Amino­adipic and 2-oxoadipic aciduria (OMIM 204750) is an inborn error of metabolism with questionable clinical consequence (Fischer *et al.*, 1974 ▸), characterized biochemically by increased urinary excretion of 2-oxoadipate and its transamination product 2-amino­adipate (Danhauser *et al.*, 2012 ▸; Duran *et al.*, 1984 ▸). Among <30 reported cases caused by autosomal recessive missense and nonsense mutations (Hagen *et al.*, 2015 ▸), p.G729R and p.R455Q are common variants. Additionally, a nonsense DHTKD1 mutation causes Charcot–Marie–Tooth disease type 2Q (CMT2Q, OMIM 615025), an autosomal dominant neurodegenerative disorder characterized by motor and sensory neuropathies (Xu *et al.*, 2012 ▸).

While structural studies have been carried out for PDH and BCKDH E1 enzymes from various organisms across the phyla, including human (Ævarsson *et al.*, 2000 ▸; Ciszak *et al.*, 2003 ▸), only prokaryotic OGDHs have been crystallized. These include the apo structure of *Escherichia coli* OGDH (ecOGDH) (Frank *et al.*, 2007 ▸), as well as various structures of *Mycobacterium smegmatis* OGDH (msOGDH) complexed with active-site catalytic intermediates (Wagner *et al.*, 2011 ▸, 2014 ▸, 2019 ▸). In this study, we report the crystal structure of human DHTKD1 (hDHTKD1) and a cryo-EM reconstruction of the human DLST (hDLST) catalytic core. We also characterize disease-causing variants of DHTKD1 for protein thermostability and interaction with DLST.

## 2. Materials and methods   

### 2.1. Expression and purification of hDHTKD1 and hDLST   

Site-directed mutations were constructed using the QuikChange mutagenesis kit (Stratagene) and confirmed by sequencing. All primers are available upon request. Wild-type (WT) and variant DHTKD1 proteins, as well as all DLST proteins, were expressed in *E. coli* BL21(DE3)R3-Rosetta cells from 1–6 l of Terrific Broth culture. Cultures were grown at 37°C until an optical density (OD_600_) of 1.0, when they were cooled to 18°C and induced with 0.1 m*M* IPTG overnight. Cultures were harvested at 4000*g* for 30 min. Cell pellets were lysed by sonication at 35% amplitude, 5 s on 10 s off, and centrifuged at 35 000*g*. The clarified cell extract was incubated with Ni-NTA resin pre-equilibrated with lysis buffer (50 m*M* HEPES pH 7.5, 500 m*M* NaCl, 20 m*M* imidazole, 5% glycerol, 0.5 m*M* TCEP). The column was washed with 80 ml binding buffer (50 m*M* HEPES pH 7.5, 500 m*M* NaCl, 5% glycerol, 20 m*M* imidazole, 0.5 m*M* TCEP) and 80 ml wash buffer (50 m*M* HEPES pH 7.5, 500 m*M* NaCl, 5% glycerol, 40 m*M* imidazole, 0.5 m*M* TCEP), and eluted with 15 ml of elution buffer (50 m*M* HEPES pH 7.5, 500 m*M* NaCl, 5% glycerol, 250 m*M* imidazole, 0.5 m*M* TCEP). The eluant fractions were concentrated to 5 ml and applied to a Superdex 200 16/60 column pre-equilibrated in GF buffer (50 m*M* HEPES pH 7.5, 500 m*M* NaCl, 0.5 m*M* TCEP, 5% glycerol). Eluted protein fractions were concentrated to 10–15 mg ml^−1^. Lipoylation of DLST proteins was verified by intact mass spectrometry.

### 2.2. Co-expression of DHTKD1 and DLST   

The DHTKD1–DLST complex used in this study was co-expressed in both *E. coli* and insect Sf9 cells. For *E. coli* co-expression, hDHTKD1_45–919_ was subcloned into the pCDF-LIC vector (incorporating a His-tag) and the resultant plasmid was co-transformed with the plasmid encoding untagged hDLST_68–453_ in the pNIC-CT10HStII vector. Co-transformed cultures were grown and protein purification was performed, as described above for DHTKD1 alone. For co-expression in insect cells, baculoviruses were produced by transformation of DH10Bac cells. Viruses were amplified by infecting Sf9 insect cells in 250 ml of sf900II serum free protein-free insect-cell medium (Thermo Fisher Scientific) and grown for 65 h at 27°C in 1 l shakers. Sf9 culture was co-infected in a 1:1 ratio with two of third-generation viruses each at 1.5 ml l^−1^. One baculovirus pFB-Bio5 vector expresses His-tagged hDHTKD1_45–919_ and the other baculovirus pFB-LIC-Bse vector expresses His-tagged hDLST_68–453_. The cultures were grown at 27°C for 72 h in 3 l flasks before harvesting at 900*g* for 30 min. The purification of Sf9 expressed proteins was carried out mostly as above. It only differed in adding 1:1000 benzonase to the lysis buffer and a gentler sonication cycle of 4 s on, 12 s off.

### 2.3. Crystallization and structure determination of DHTKD1   

Crystals were grown by the vapour-diffusion method. To crystallize hDHTKD1_45–919_, concentrated protein was incubated for 30 min on ice with 3 m*M* MgCl_2_ and 3 m*M* ThDP before being centrifuged for 10 min at 13 500*g* to remove any precipitation. Sitting drops containing 75 nl of protein (10 mg ml^−1^) and 75 nl of well solution containing 20%(*w*/*v*) PEG 3350, 0.1 *M* bis-tris-propane pH 8.5, 0.2 *M* sodium formate and 10%(*v*/*v*) ethyl­ene glycol were equilibrated at 4°C. Crystals were mounted and frozen without additional cryo-protectant, as the crystallization condition contains 10%(*v*/*v*) ethyl­ene glycol. Diffraction data were collected at the Diamond Light Source beamline I03 and processed using the *CCP*4 program suite (Winn *et al.*, 2011 ▸). hDHTKD1_45–919_ crystallized in the primitive space group *P*1 with two molecules in the asymmetric unit. The structure was solved by molecular replacement using the program *Phaser* (McCoy *et al.*, 2005 ▸) and the *E. coli* OGDH structure (PDB code 2jgd; Frank *et al.*, 2007 ▸) as the search model. The structure was refined using *Phenix* (Adams *et al.*, 2010 ▸), followed by iterative cycles of model building in *Coot* (Emsley & Cowtan, 2004 ▸). Statistics for data collection and refinement are summarized in Table 1 ▸. Protein interfaces were analysed with the software *PISA* (Krissinel & Henrick, 2007 ▸).

### 2.4. DHTKD1 enzyme assay   

The enzymatic activity assay was performed in triplicates, in a buffer containing 35 m*M* potassium phosphate (KH_2_PO_4_), 0.5 m*M* EDTA, 0.5 m*M* MgSO_4_, 2 m*M* 2OA or 2OG, 1 m*M* ThDP, 5 m*M* sodium azide (NaN_3_) and 60 µ*M* 2,6-dichloro­phenolindophenol (DCPIP), pH 7.4. The activity was determined as a reduction of DCPIP at λ = 610–750 nm, 30°C (Sauer *et al.*, 2005 ▸), with and without 2OA or 2OG. The dye DCPIP changes colour from blue to colourless when being reduced (VanderJagt *et al.*, 1986 ▸). To obtain *K*_m_ and *V*_max_, different concentrations of 2OA (0.1, 0.05, 0.1, 0.25, 0.5, 0.75, 1 and 2 m*M*) and no substrate were measured in a 96-well microtitre plate (total well volume = 300 µl). The ensuing OD values were plotted on a graph (slope = 1/*V*_max_; *Y* intercept = *K*_m_/*V*_max_) to calculate *K*_m_ and *V*_max_ using the Hanes Woolf plot: 
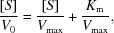
where *V*_0_ = initial velocity, [*S*] = substrate concentration and *V*_max_ = maximum velocity.

### 2.5. Small-angle X-ray scattering   

Small-angle X-ray scattering (SAXS) experiments were performed at a wavelength of 0.99 Å at the Diamond Light Source beamline B21 coupled to the appropriate size-exclusion column (Harwell, UK) and equipped with a PILATUS 2M 2D detector at a distance of 4.014 m from the sample, 0.005 < *q* < 0.4 Å^−1^ (*q* = 4π sin θ/λ, 2θ is the scattering angle). hDHTKD1_45–919_ at 20 mg ml^−1^ in 10 m*M* HEPES-NaOH pH 7.5, 200 m*M* NaCl, 0.5 m*M* TCEP and 2% glycerol was applied onto a Shodex KW404-4F column. hDHTKD1_45–919_ co-expressed with hDLST_68–453_ in baculo Sf9 cells at 5 mg ml^−1^ in 25 m*M* HEPES pH 7.5, 200 m*M* NaCl, 0.5 m*M* TCEP, 2% glycerol and 1% sucrose was applied onto the Shodex KW404-4F column. hDLST at 10 mg ml^−1^ in 25 m*M* HEPES pH 7.5, 200 m*M* NaCl, 0.5 m*M* TCEP, 2% glycerol and 1% sucrose was applied onto a Shodex 405-4F column.

SAXS measurements were performed at 20°C using an exposure time of 3 s frame^−1^. SAXS data were processed and analyzed using the *ATSAS* program package (Franke *et al.*, 2017 ▸) and *Scatter* (http://www.bioisis.net/scatter). The radius of gyration *R*_g_ and forward scattering *I*(0) were calculated by Guinier approximation. The maximum particle dimension *D*_max_ and *P*(*r*) function were evaluated using the program *GNOM* (Svergun, 1992 ▸).

### 2.6. Solution analysis   

Analytical gel filtration was performed on a Superdex 200 Increase 10/300 GL column or Superose 6 Increase 10/300 GL (GE Healthcare) pre-equilibrated with 20 m*M* HEPES pH 7.5, 150 m*M* NaCl and 0.5 m*M* TCEP.

### 2.7. Differential scanning fluorimetry (DSF)   

DSF was performed in a 96-well plate using an Mx3005P RT PCR machine (Stratagene) with excitation and emission filters of 492 and 610 nm, respectively. Each well (20 µl) consisted of protein (2 mg ml^−1^ in 100 m*M* HEPES pH 7.5, 150 m*M* NaCl, 5% glycerol), SYPRO Orange (Invitrogen, diluted 1000-fold of the manufacturer’s stock). Fluorescence intensities were measured from 25 to 96°C with a ramp rate of 1°C min^−1^. *T*_m_ was determined by plotting the intensity as a function of temperature and fitting the curve to a Boltzmann equation. Temperature shifts, Δ*T*_m_, were determined as described (Niesen *et al.*, 2007 ▸) and final graphs were generated using *GraphPad Prism* (v.7; *GraphPad* software). Assays were carried out in technical triplicate.

### 2.8. *MIDAS* protein–metabolite screening   

Protein–metabolite interaction screening using an updated *MIDAS* platform was performed similar to Orsak *et al.* (2012 ▸). Briefly, a flow-injection analysis mass-spectrometry (FIA-MS) validated library of 412 metabolite standards was combined into four defined screening pools in 150 m*M* ammonium acetate pH 7.4. For each metabolite pool, 5 µl of target protein was arrayed in triplicate across a SWISSCI 10 MWC 96-well microdialysis plate (protein chambers). To the trans side of each dialysis well, 300 µl of a 50 µ*M* metabolite pool supplemented with 1 m*M* ThDP and 1 m*M* MgCl_2_ was arrayed in triplicate per hDHTKD1_45–919_ protein (metabolite chambers). Dialysis plates were placed in the dark at 4°C on a rotating shaker (120 rev min^−1^) and incubated for 40 h. Post-dialysis, protein- and metabolite-chamber dialysates were retrieved, normalized and diluted 1:10 in 80% methanol, incubated for 30 min on ice, and centrifuged at 3200*g* RCF for 15 min to remove precipitated protein. Analytes were aliquoted across a 384-well microvolume plate and placed at 4°C in a Shimadzu SIL-20ACXR autosampler for FIA-MS analysis. Then, 2 µl of each sample was analysed in technical triplicate by FIA-MS on a SCIEX X500R QTOF MS with interspersed injections of blanks.

### 2.9. *MIDAS* data analysis   

FIA-MS spectra collected from *MIDAS* protein–metabolite screening was qualitatively and quantitatively processed in *SCIEX* OS 1.5 software to determine relative metabolite abundance by integrating the mean area under the curve across technical triplicates. Log2(fold change) for each metabolite was calculated from the relative metabolite abundance in the protein chamber (numerator) and metabolite chamber (denominator) from dialysis triplicates. For each technical triplicate, up to one outlier was removed using a *z*-score cutoff of five (<0.1% of observations). The corrected technical replicates were collapsed to one mean fold-change summary per protein–metabolite pair. To remove fold-change variation that was not specific to a given metabolite–protein pair, the first three principal components of the cumulative screening dataset were removed (∼75% of observed variance) creating Log2(corrected fold change). Protein–metabolite *z* scores were determined by comparing the target protein–metabolite Log2(corrected fold change) to a no-signal model for that metabolite using measures of the central tendency (median) and standard deviation (extrapolated from the 25–75% quantiles) which are not biased by the signals in the tails of a metabolite’s fold-change distribution. *z* scores were false-discovery rate controlled using Storey’s *q* value (http://github.com/jdstorey/qvalue). Protein–metabolite interactions with *p* values < 0.05 and *q* values < 0.1 were considered significant.

### 2.10. Grid preparation and EM data collection   

3 µl of 0.4 mg ml^−1^ purified complex from *E. coli* or Sf9 cells were applied to the glow-discharged Quantifoil Au R1.2/1.3 grid (Structure Probe). Blotting and vitrification in liquid ethane was carried out using a Vitrobot Mark IV (FEI Company) at 4°C and 95% humidity with a 9 s wait and a 3 s blot at zero blotting force from both sides. Cryo grids were loaded into a Glacios transmission electron microscope (ThermoFisher Scientific) operating at 200 keV with a Falcon3 camera. For the *E. coli* sample, three screening images were recorded in linear mode with a pixel size of 0.96 Å and a defocus set to −3 µm. The Sf9 sample was also recorded in linear mode with a pixel size of 0.96 Å and a defocus range of −1 to −3.1 µm (steps of 0.3 µm). Data were collected with a total dose of 32.52 e Å^−2^ and images were recorded with a 1 s exposure over 19 frames. Details are summarized in Table S1 in the Supporting information. Representative micrographs of both datasets are shown in Figs. S9(*a*) and S9(*b*) in the Supporting information.

### 2.11. EM data processing   

The three single-frame micrographs from the *E. coli* dataset were used for manual picking after contrast transfer function (CTF) parameters were determined by *CTFFIND*4.1 (Rohou & Grigorieff, 2015 ▸). A total of 572 particles were extracted with a box size of 344 pixels, and 2D classification was performed. A total of five classes containing 272 particles were compared with an equivalent set of particles derived from the Sf9 dataset [Fig. S9(*c*)]. The full data-processing workflow for the Sf9 derived complex is illustrated in Fig. S10. A total of 619 dose-fractioned movies were corrected for drift using *RELION’s MotionCor2* (Zheng *et al.*, 2017 ▸) with the dose-weighting option. CTF parameters were determined by *CTFFIND*4.1 (Rohou & Grigorieff, 2015 ▸). A subset of 1244 particles were manually picked and extracted with a box size of 344 pixels rescaled to 172 pixels. Eight classes were selected from one round of 2D classification for reference-based autopicking using *RELION* 3.0 (Scheres & Chen, 2012 ▸). 165 739 particles were extracted with a box size of 344 pixels rescaled to 172 pixels. All downstream particle classification, refinement and post-processing steps were performed in *RELION* 3.0 (Scheres & Chen, 2012 ▸). Junk particles were removed using 2D classification. Parallel rounds of 3D classification with (O) and without octahedral symmetry (C1) revealed no classes with additional density visible beyond the C-terminal catalytic domain. Reclassification with a soft mask also resulted in classes with no apparent density for the DLST N terminus. Subsequently, masked 3D refinement with the highest-resolution class from the symmetry-imposed masked 3D classification resulted in a 5 Å map. Finally, a further round of masked 3D classification without alignment and a regularization parameter *T* value of 20 was used to identify classes with the highest-resolution features. The resulting 3356 particles were refined to a global resolution of 4.7 Å based on the Fourier shell correlation (FSC) 0.143 threshold criterion. The orientation distribution of the final map was visualized with *Chimera*. Local resolution was calculated with *RELION* 3.0 (Scheres & Chen, 2012 ▸).

### 2.12. EM model building and refinement   

Model building and refinement was carried out using the suite of programs in *CCP-EM* (Burnley *et al.*, 2017 ▸). To fit a template to the final map the *E. coli* DLST orthologue structure (PDB code 1scz; Schormann *et al.*, unpublished work) was used. The sequence was humanized and residues truncated to the alpha carbon using *CHAINSAW* (Stein, 2008 ▸). The oligomeric structure was docked into the density map, sharpened with a *B* factor of −281 Å^2^, using *MOLREP*. One round of refinement using *REFMAC*5 was carried out with *ProSMART* restraints generated from the *E. coli* DLST orthologue to avoid overfitting. Figures displaying model fit to density were made using *Chimera* (Pettersen *et al.*, 2004 ▸). Overfitting was monitored through simultaneous refinement against the two half maps from the final 3D refinement. FSC between map and model was calculated using model validation in *CCP-EM* (Burnley *et al.*, 2017 ▸).

## 3. Results and discussion   

### 3.1. Overall structure of hDHTKD1 homodimer   

**Figure 1:**
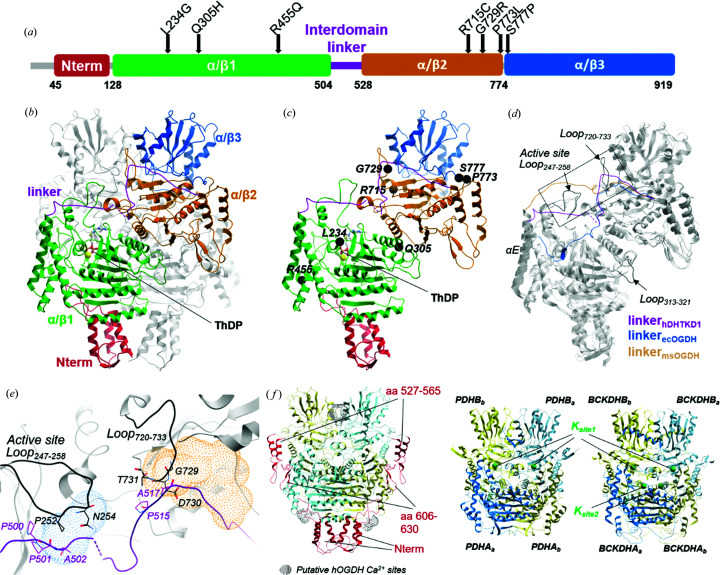
Overall structure of hDHTKD1. (*a*) A schematic of hDHTKD1 domain organization, indicating disease-causing missense mutations with arrows. (*b*) Structure of the hDHTKD1 homodimer, where one subunit is coloured according to the domain organization from panel (*a*) and the other subunit is coloured grey. ThDP co-factors and Mg^2+^ ions are shown as sticks and spheres, respectively. (*c*) Sites of missense mutations are shown as black spheres on one hDHTKD1 protomer [same view as panel (*b*)]. (*d*) Structural superposition of hDHTKD1, ecOGDH (PDB code 2jgd) and msOGDH (PDB code 6r2b; Wagner *et al.*, 2019 ▸) highlighting their different inter-domain linkers (coloured purple, blue and light brown, respectively). (*e*) A magnified view of the dotted box in panel (*d*), showing how the hDHTKD1 inter-domain linker (purple) packs against two loop regions (black). Binding positions for the allosteric effectors AMP in the ecOGDH structure (PDB code 2jgd) and acetyl-CoA in the msOGDH structure (PDB code 2y0p; Wagner *et al.*, 2011 ▸) are shown as blue and brown meshes, respectively. (*f*) Overall architectures of the hDHTKD1 homodimer (left), the human (PDHA–PDHB)_2_ heterotetramer (middle, PDB code 1ni4; Ciszak *et al.*, 2003 ▸) and the human (BCKDHA–BCKDHB)_2_ heterotetramer (right, PDB code 1dtw; Ævarsson *et al.*, 2000 ▸) are shown. hDHTKD1 homodimer has a larger volume because of the insertions coloured red.

hDHTKD1 is a 919-amino acid (aa) polypeptide [Fig. 1 ▸(*a*)], with the N-terminal 22 aa predicted to form the mitochondrial targeting signal peptide (Bunik & Degtyarev, 2008 ▸). We expressed in *E. coli* the soluble proteins for the precursor hDHTKD1_1–919_, the predicted mature protein (hDHTKD1_23–919_), as well as a further truncated construct (hDHTKD1_45–919_) removing the putatively disordered aa 24–44 (Fig. S1). Despite various attempts, only the construct hDHTKD1_45–919_ yielded crystals, upon pre-incubation with ThDP and Mg^2+^ prior to crystallization trials. This protein construct is active *in vitro*, exhibiting E1 de­carboxyl­ase activity with 2OA as substrate in a colorimetric assay using 2,6-dichlorphenol indophenol as reductant (*V*_max_ = 14.2 µmol min^−1^ mg^−1^ protein, *K*_m, 2OA_ = 0.2 m*M*; see Section 2.4).

The crystal structure of hDHTKD1_45–919_ is determined to 1.9 Å resolution by molecular replacement, using the *E. coli* OGDH structure (PDB code 2jgd, 38% sequence identity; Frank *et al.*, 2007 ▸) as the search template (Table 1 ▸). The asymmetric unit contains two DHTKD1 protomers [A and B; Fig. 1 ▸(*b*)] arranged as an intertwined obligate homodimer in a similar manner to OGDH, burying a large 5600 Å^2^ (18%) area of monomeric accessible surface at the dimer interface. This crystal homodimer is consistent with SAXS analysis of hDHTKD1_45–919_ protein in solution, with the theoretical scattering curve of the dimer displaying a good fit to experimental data (χ^2^ of 3.8, Fig. S2).

Our DHTKD1 structural model [Fig. 1 ▸(*c*)] consists of residues 53–915 from both chains, with the exception that no electron density was observed for two surface exposed loop regions (aa 274–275_chain A_/274–277_chain B_ and aa 502–508_chain A_/505–508_chain B_). DHTKD1 is structurally composed of an N-terminal helical bundle (aa 53–127) followed by three α/β domains (α/β1, aa 129–496, Pfam PF00676; α/β2, aa 528–788, PF02779; and α/β3, aa 789–915, PF16870). These four structural regions assemble into two halves, inter-connected by an extended linker (aa 497–527) that threads along the protein surface [Figs. 1 ▸(*b*) and 1 ▸(*c*)].

### 3.2. Structural comparison of DHTKD1 with other E1 enzymes   

As expected, a *DALI* search (Holm & Sander, 1995 ▸) reveals that the closest structural homologue to hDHTKD1 is msOGDH [*z* score 55.7, root-mean-square deviation (RMSD) of 1.9 Å and 38% sequence identity] and ecOGDH (*z* score 50.3, RMSD of 1.8 Å and 40% sequence identity). The main structural divergence is found in their interdomain linkers, which traverse the respective protein surface via different trajectories [Fig. 1 ▸(*d*)]. Importantly, the hDHTKD1 linker (498–527) packs against two loop regions that are longer than the equivalents in ecOGDH and msOGDH [Fig. 1 ▸(*e*)]. These include the ‘active site loop’ (aa 247–258), and a DHTKD1-unique region (aa 720–733) identified as ‘Δ3’ in the work of Bunik & Degtyarev (2008 ▸) (Fig. S3). The path traversed by the hDHTKD1 linker, not an artefact from crystal packing (Fig. S4), also overlaps with the binding sites for the allosteric activators of ecOGDH [acetyl-CoA, (Frank *et al.*, 2007 ▸)] and msOGDH [AMP, (Wagner *et al.*, 2011 ▸)] revealed from their structures [Fig. 1 ▸(*e*), meshes]. These allosteric sites are probably not present in DHTKD1 structure because of low sequence conservation in the neighbourhood (Fig. S3).

To a lesser degree, hDHTKD1 is also structurally homologous to the E1 enzymes of human PDH and BCKDH (also known as 2-oxoisovalerate de­hydrogenase) [*z* score 30, RMSD of 3.0–3.5 Å and 16% sequence identity (Ævarsson *et al.*, 2000 ▸; Ciszak *et al.*, 2003 ▸)], which are heterotetramers built from two copies of two subunits [Fig. 1 ▸(*f*)]. This contrasts with DHTKD1, OGDH and presumably OGDHL, which are homodimers. PDH and BCKDH form a more compact shape, lacking several surface insertions to the α/β core that are unique to the DHTKD1/OGDH/OGDHL subgroup. These include the helical bundle at the N terminus, and the β- and helical hairpins (aa 527–565, 606–630) within the α/β2 domain [Fig. 1 ▸(*f*), red ribbons]. PDH and BCKDH structures also contain K^+^ binding sites that play a role in enzymatic regulation [Fig. 1 ▸(*f*), green spheres]. We did not observe any difference density that suggests metal binding in the equivalent region of DHTKD1. Metal-dependent regulation is also featured in mammalian OGDH enzymes (Rutter *et al.*, 1989 ▸; Lawlis & Roche, 1981 ▸), mediated by Ca^2+^ binding motifs unique to the OGDH N terminus and a region equivalent to the DHTKD1 Δ3 (Rigden & Galperin, 2004 ▸). Again, these motifs are not present in prokaryotic OGDHs or DHTKD1.

### 3.3. The DHTKD1 active site favours 2OA as substrate   

Each DHTKD1 subunit in the crystal homodimer is bound with a ThDP co-factor [Figs. 1 ▸(*b*) and 1 ▸(*c*)], at a site formed from both subunits (Fig. S5). The ThDP pyrophosphate moiety binds to the α/β1 domain of one subunit, the pyrimidine ring binds to the α/β2 of the other subunit, while the central thia­zolium ring sits between the subunits. The ThDP binding residues are highly conserved among OGDH and E1 homologues (Fig. S3). These include Asp333 and Asn366 which bridge the ThDP pyrophosphates with the Mg^2+^ ion. Also, Leu290 acts as a hydro­phobic wedge to form the characteristic V-shaped conformation of ThDP, bringing the N4′ amino group of the pyrimidine ring into close proximity (3.08 Å) with the C2 proton of the thia­zolium ring. Essential for catalysis, Glu640 triggers a proton relay to activate the co-factor into a reactive ylide (Fig. S5). Reaction then ensues via a nucleophilic attack by the ThDP ylide on the keto carbon of the substrate, forming a pre-de­carboxyl­ation intermediate that is in turn de­carboxyl­ated into an enamine-like ThDP adduct.

**Figure 2:**
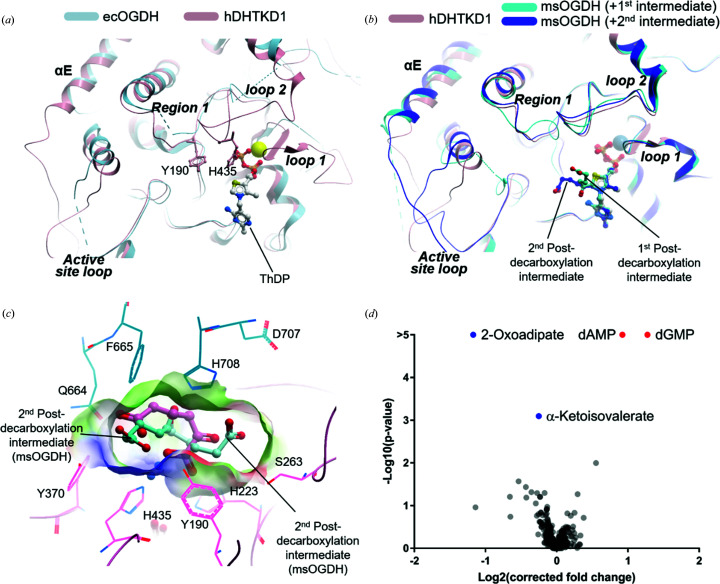
Substrate binding pocket of hDHTKD1. (*a*) A view of the active site from the hDHTKD1 structure (pink ribbon) bound with ThDP (sticks, white carbon atoms) and Mg^2+^ (yellow), overlaid with the ecOGDH structure in the apo form (light blue ribbon; PDB code 2jgd), to highlight structural differences in various loop regions. (*b*) The same hDHTKD1 active-site view as in panel (*a*), overlaid with msOGDH structures in complex with the first post-de­carboxyl­ation intermediate (cyan ribbons for proteins, sticks with cyan carbon atoms for ligands; PDB code 3zht) and second post-de­carboxyl­ation intermediate (blue ribbons for proteins, sticks with blue carbon atoms for ligands; PDB code 3zhu). (*c*) The hDHTKD1 substrate pocket is lined by residues from both subunits of the homodimer (pink and blue lines). Overlaid in this pocket is the putative 2OA ligand (pink sticks) modelled in our density (Fig. S6) and the post-de­carboxyl­ation intermediates (cyan sticks) bound to the msOGDH structures. (*d*) The hDHTKD1–metabolite interactome as determined by *MIDAS*. hDHTKD1 significantly depleted 2OA and α-oxoisovalerate, and enriched dAMP and dGMP. The cutoff for significance was *p* < 0.05 and *q* < 0.1.

Compared with an apo E1 structure such as that of ecOGDH, our ThDP-bound DHTKD1 structure highlights four loop segments in the active site that undergo disorder-to-order transition during co-factor binding [Fig. 2 ▸(*a*)]. Using nomenclature from the work of Bunik & Degtyarev (2008 ▸) (Fig. S3), these include: ‘Region 1’ (aa 187–195), which contributes Tyr190 to the substrate binding site; the active site loop (aa 247–258), with different length and sequence from OGDHs; ‘loop 1’ (aa 366–383), which contributes the L_368_GY_370_ motif to bind the ThDP pyrophosphate; and ‘loop 2’ (aa 434–445), which contributes residues to engage with the E2 enzyme for acyl­transfer (*e.g.* His435). The conformations seen in our holo structure are similar to those of msOGDH structures bound with the post-de­carboxyl­ation co-factor conjugates (Wagner *et al.*, 2014 ▸) [Fig. 2 ▸(*b*)].

In one X-ray dataset, we observed OMIT map electron density at one active site of the homodimer that is not accounted for by the co-factor or any component of the crystallization condition [Fig. S6(*a*)]. This density is adjacent to but disjointed from the ThDP co-factor [Fig. S6(*b*)], at a location partly overlapping the two conformations of post-de­carboxyl­ation intermediate seen in the msOGDH structures (PDB codes 3zht and 3zhu; Wagner *et al.*, 2014 ▸) [Fig. 2 ▸(*b*)]. The size of this density feature can accommodate a C6 ligand such as 2OA without covalent linkage to ThDP. Although the observed ligand, likely co-purified with the protein, did not undergo enzymatic turnover, the keto carbon can be placed at 3.5 Å from the ThDP thia­zolium C2 and hence be compatible with the nucleophilic attack and subsequent de­carboxyl­ation [Fig. S6(*c*)]. While in good agreement with OMIT map, the 2OA model was not included in the deposited structure, in light of no further experimental evidence of its presence.

DHTKD1 and OGDH overlap to some extent in their *in vitro* reactivity towards 2OG and 2OA (Nemeria, Gerfen, Yang *et al.*, 2018 ▸; Leandro *et al.*, 2019 ▸). For example, soaking msOGDH crystals with 2OA and 2OG both yielded similar post-de­carboxyl­ation intermediates (Wagner *et al.*, 2014 ▸). Nevertheless, hDHTKD1 turns over 2OA with 40-fold higher catalytic efficiency than over 2OG (Nemeria, Gerfen, Yang *et al.*, 2018 ▸). The hDHTKD1 active site reveals several amino acids poised to interact with the substrate, which are not conserved with OGDH orthologues. Two of them involve substitution to more polar residues *i.e.* Tyr190 (from Phe_OGDH_) and Tyr370 (Phe_OGDH_), while the other two involve substitution to less bulky residues *i.e* Ser263 (Tyr_OGDH_) and Asp707 (Glu_OGDH_) (Fig. S3). Overlaying the two msOGDH post-de­carboxyl­ation intermediates [first and second conformers, sticks in Fig. 2 ▸(*b*); Wagner *et al.* (2014 ▸)] onto the hDHTKD1 substrate pocket clearly explained how these substituted amino acids can stabilize catalytic intermediates generated from the longer 2OA substrate [Fig. 2 ▸(*c*)]. The 2OA terminal carboxyl group from the first conformer in msOGDH (PDB code 3zht) can be sandwiched between hDHTKD1 Tyr190 (Phe_OGDH_) and Tyr370 (Phe_OGDH_) to form polar interactions, while hDHTKD1 Ser263 (Tyr_OGDH_) and Asp707 (Glu_OGDH_) increase pocket volume to accommodate the terminal carboxyl group from the second conformer in msOGDH (PDB code 3zhu). It remains to be determined whether the post-de­carboxyl­ation intermediate of hDHTKD1 also exists in dual conformation. One can rationalize that the DHTKD1 substrate pocket is engineered to accommodate the slightly larger and more polar 2OA substrate, providing a structural basis for its superior catalytic efficiency over 2OG.

### 3.4. DHTKD1 preferentially interacts with 2OA in solution   

We further explored the substrate preference of hDHTKD1 by mapping its metabolite interactome using *MIDAS*, a mass spectrometry-based equilibrium dialysis approach (Orsak *et al.*, 2012 ▸). From a screening library of 412 human metabolites, 2OA was observed as the most significant (*p* < 4.33 × 10^−54^, *q* < 2.59 × 10^−51^) interaction with hDHTKD1_45–919_ in the presence of ThDP and Mg^2+^ [Fig. 2 ▸(*d*), see Supplementary File S1 in the Supporting information]. Furthermore, 2OA had the most negative Log2(corrected fold change) value (−1.17), suggesting that hDHTKD1 enzymatically processed 2OA during the *MIDAS* screening. Relative to 2OA, the 2OG interaction with hDHTKD1 was not significant (*p* < 0.17, *q* < 0.74) and had a relatively small negative Log2(corrected fold change) value (−0.28). The higher confidence and fold change observed for 2OA, relative to 2OG, are in complete agreement with the substrate preference of hDHTKD1.

α-Ketoisovalerate (also known as α-oxoisovalerate), the primary product of valine degradation by branched-chain-amino-acid amino­transferases, was the second most significant metabolite (*p* < 8.00 × 10^−4^, *q* < 2.06 × 10^−2^) and had the second most negative Log2(corrected fold change) value (−0.25), suggesting hDHTKD1 could interact with and may also enzymatically process α-oxoisovalerate. The de­oxy­purine monophosphates, dAMP and dGMP, had significant (*p* < 4.47 × 10^−11^, *q* < 4.84 × 10^−9^ and *p* < 5.13 × 10^−18^, *q* < 1.07 × 10^−15^, respectively) positive Log2(corrected fold change) values (0.92 and 1.27), suggesting binding to hDHTKD1. These results support observations that purine nucleotides functionally regulate eukaryotic OGDHc (Lawlis & Roche, 1981 ▸; Craig & Wedding, 1980 ▸) and perhaps OADHc. Further experiments are warranted to understand the functional relevance of α-oxoisovalerate and nucleotide mono­phosphates on DHTKD1 activity.

### 3.5. DHTKD1 and DLST form direct interactions   

There is literature evidence that the E1 and E2 components of 2-oxoacid de­hydrogenase complexes interact directly as a binary subcomplex, in the absence of E3 (Zhou *et al.*, 2018 ▸; Park *et al.*, 2004 ▸; Patel *et al.*, 2009 ▸). For some E1 enzymes such as OGDH and PDH, the N terminus is known to be important for the direct interaction with E2 (Zhou *et al.*, 2018 ▸; Park *et al.*, 2004 ▸) and E3 (McCartney *et al.*, 1998 ▸), although this region is notably different for DHTKD1. For example, the hDHTKD1 precursor encodes a mere 50-aa segment before the first α-helix of the structure, while the hOGDH equivalent region is longer (121 aa) and contains two DLST-binding motifs (Zhou *et al.*, 2018 ▸) not preserved in DHTKD1 [Fig. S1(*b*)]. This suggests that the manner in which DHTKD1 and OGDH (E1) interact with DLST (E2) could be different.

**Figure 3:**
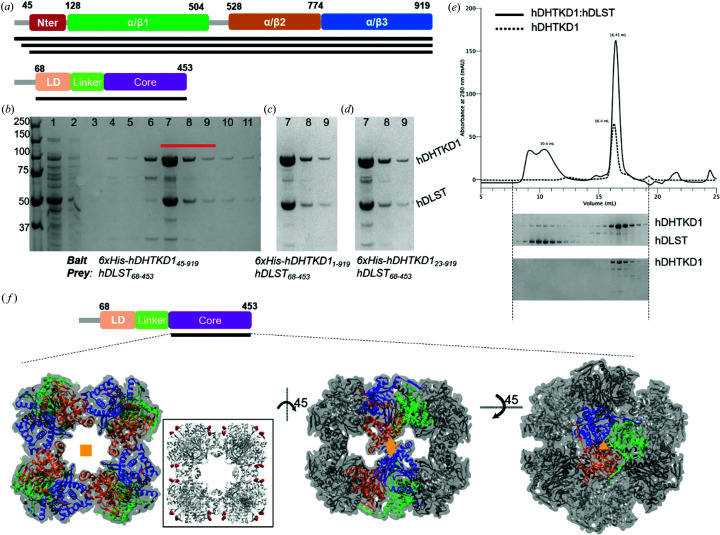
Interaction studies of hDHTKD1 and hDLST. (*a*) Constructs of hDHTKD1 and hDLST (black bars) used in the affinity pulldown experiments. (*b*)–(*d*) SDS–PAGE showing affinity pulldown of untagged hDLST_68–453_ from (*b*) His_6_-tagged hDHTKD1_45–919_, (*c*) hDHTKD1_1–919_ and (*d*) hDHTKD1_23–919_. The original uncropped SDS–PAGE gels are shown in Fig. S7. For panel (*b*), the lanes loaded are: 1, flow-through; 2–6, wash fractions of increasing imidazole concentration; and 7–11, elution fractions with 250 m*M* imidazole. SDS–PAGE gel fragments from panels (*c*) and (*d*) show only the first three elution fractions *i.e.* lanes 7, 8 and 9 [equivalent to the lanes marked in red in panel (*b*)]. (*e*) Chromatogram and SDS–PAGE from SEC runs of hDHTKD1_45–919_ protein alone (dashed line) and of hDHTKD1_45–919_ co-expressed with hDLST_68–453_. Elution volumes for the complex peak and hDHTKD1-alone peak are shown. (*f*) An EM map of the hDLST 24-mer catalytic core overlaid with a humanized model of *E. coli* DLST (PDB code 1scz). The three views show how the trimer building block (monomers shown as blue, orange and green ribbons) is assembled into the 24-mer core via fourfold (left), twofold (middle) and threefold (right) symmetry axes, respectively. Inset of the left view shows how the first residue (aa 219, red spheres) from each of the 24 hDLST catalytic cores is distributed at the surface of the cube structure.

hDLST as a precursor protein is structurally composed of [Fig. 3 ▸(*a*)]: the mitochondrial target sequence (aa 1–67), the N-terminal single lipoyl domain (aa 68–154) to which a di­hydro­lipoyl moiety is covalently attached through a lysine residue (Lys110), the C-terminal catalytic domain responsible for the multimeric assembly and harbouring the acyl-transferase active site (aa 211–453), and the flexible inter-domain linker (aa 155–210). We opted to reconstitute the DHTKD1–DLST binary complex by co-expressing His-tagged hDHTKD1_45–919_ and hDLST_68–453_ in *E. coli* followed by affinity chromatography. Untagged hDLST_68–453_ was found to co-purify with His-tagged hDHTKD1_45–919_ immobilized on Ni affinity resin [Fig. 3 ▸(*b*)], and to a similar extent with hDHTKD1_1–919_ [Fig. 3 ▸(*c*)] and hDHTKD1_23–919_ [Figs. 3 ▸(*d*), S7(*a*), S7(*b*) and S7(*c*)]. Hence the hDHTKD1 N-terminal 45 aa, not present in our structural model and replacing the DLST-binding motifs mapped for hOGDH, does not play a role in the DHTKD1–DLST interaction. Size-exclusion chromatography (SEC) using an analytical Superose 6 Increase column eluted the complex at *V*_e_ = 10.4 ml (*V*_e_ = elution volume), as compared with hDHTKD1_45–919_ protein alone which eluted later at *V*_e_ = 16.3 ml [Fig. 3 ▸(*d*)]. Our attempts to mix the binary DHTKD1–DLST complex with purified DLD did not yield a stable three-way complex in SEC [Fig. S7(*d*)], as was the case shown for OGDHc previously (Zhou *et al.*, 2018 ▸).

Similar DHTKD1–DLST complex can also be formed by co-expression in the baculo Sf9 cells. When expressed alone in Sf9, the hDLST_68–453_ protein is highly prone to degradation, with a significant proportion fragmenting into two halves [Figs. S8(*a*) and S8(*b*)]. When the DHTKD1 and DLST proteins are co-expressed, hDHTKD1_45–919_ co-purified in SEC together with both the hDLST_68–453_ intact protein and the C-terminal fragment (containing the catalytic core), while the N-terminal fragment (containing the lipoyl domain and linker) was not part of this complex [Fig. S8(*c*)]. This suggests that the DLST N-terminal fragment alone is not sufficient to interact with DHTKD1, although this DLST region was previously mapped to be interacting with the binding motifs at the hOGDH-unique N terminus (Zhou *et al.*, 2018 ▸).

Altogether, our data reinforce the notion that DHTKD1 and OGDH interact with DLST differently despite the structural conservation. This difference is not surprising considering these enzymes are present in distinct cellular contexts. While DHTKD1 is responsible for the last step of lysine and tryptophan catabolism, OGDHc operates as a rate-limiting step in the Krebs cycle. Therefore, they are expected to be regulated by different mechanisms, both spatially (*e.g.* by employing distinct binding partners, co-factors and post-translational modifications) and temporally (*e.g.* by displaying different affinity/kinetics towards binding partners/co-factors).

### 3.6. Insight into complex assembly from cryo-EM and SAXS studies   

To provide a structural context for the DHTKD1–DLST interactions, we attempted single-particle cryo-EM on the reconstituted binary complexes co-expressed in *E. coli* and Sf9 cells. Electron micrographs displayed the characteristic cubic cage structures of approximate dimensions 130 × 130 × 130 Å (Fig. S9), as observed for *E. coli* DLST (Knapp *et al.*, 1998 ▸) and other E2 enzymes such as *Azotobacter vinelandii* PDH E2 (Mattevi *et al.*, 1992 ▸) and bovine BCKDH E2 (Kato *et al.*, 2006 ▸).

We collected a dataset from the Sf9 co-expressed complex and generated a 3D reconstruction at 4.7 Å global resolution derived from 3356 particles (Figs. S10, S11 and Table S1). Local resolution analysis reveals a range between 4.7 and 6.3 Å [Figs. S11(*a*) and S11(*c*)]. The low resolution may in part be explained by orientation bias along the fourfold symmetry axis [Fig. S11(*b*)]. The EM reconstruction shows 24 DLST C-terminal catalytic domains assembled as eight trimer building blocks into a cubic cage with octahedral symmetry [Fig. 3 ▸(*f*)] and allows tracing of a humanized DLST model (aa 219–453 of hDLST) based on the *E. coli* structures [PDB codes 1e2o and 1scz; 60% identity; Albert *et al.* (2000 ▸); Knapp *et al.* (1998 ▸)] [Fig. S11(*f*)].

Considering the sequence conservation, the catalytic cores of *E. coli* and hDLST display essentially identical topology and symmetry along two-, three- and fourfold axes [Fig. 3 ▸(*f*)]. In this assembly, all 24 C-terminal catalytic domains have their first residue (aa 219) exposed to the surface of the core [Fig. 3 ▸(*f*), inset], presumably projecting the adjacent inter-domain linker outwards from the core in order to deliver the N-terminal lipoyl domain for engagement with E1 and E3. After processing the dataset with extensive 3D classification comparisons with and without imposing symmetry (Fig. S10), there is unfortunately no discernible density for further regions of DLST (*e.g.* N-terminal lipoyl domain) or for the DHTKD1 protein. It is likely that the DLST lipoyl domain and much of the linker region are highly flexible and dynamic, in agreement with previous attempts to structurally characterize other full-length E2 enzymes such as human PDH E2 (Yu *et al.*, 2008 ▸). Additionally, the DHTKD1–DLST inter­action could be short lived, as shown for other E1–E2 complexes. It is also possible that this region could be partially denatured by the air–water interface.

We also subjected the *E. coli* co-expressed complex to cryo-EM and observed more heterogeneous particles on the micrograph, some of which reveal extra density emanating from the cubic core to ∼10–20 Å [Fig. S9(*a*)]. This contrasts with the aforementioned Sf9 co-expressed complex, where particles are predominantly homogenous and contain only cubic cages [Fig. S9(*b*)]. Five 2D classes comprising of 272 particles from the *E. coli* co-expressed complex (manually picked across three screening micrographs and representing 48% of the maximum particles available) were compared with 2D classes with an equivalent number of particles picked from the large dataset of the Sf9 co-expressed complex [Fig. S9(*c*)]. Again, loosely defined and heterogeneous density is visible at the corners of the cubic core within the *E. coli* co-expressed complex, whereas this extra density is missing or lost through 2D classification of the equivalent Sf9 co-expressed complex. We reasoned that the additional density protruding from the core represents an ordered segment of the linker region and perhaps in some instances engages with the interaction partner DHTKD1. However, a larger high-resolution dataset of the *E. coli* complex would be required to discern the exact contribution of this additional density.

The positioning of 24 lipoyl domains at the exterior of the DLST core implies that they are all potentially available for engagement with E1 and E3. To explore the underlying stoichiometry for the DHTKD1–DLST interaction, we characterized the Sf9 co-expressed binary complex using SEC-SAXS (Fig. S12). The molecular weight (MW) of hDHTKD1_45–919_:hDLST_68–453_ derived from SAXS porod volume is 2.45 MDa, which is in close agreement with a MW of 2.7 MDa calculated for 24 × DLST and 16 × DHTKD1 protomers, assuming a stoichiometry of one DHTKD1 dimer per DLST trimer building block as suggested previously for hOGDHc (Zhou *et al.*, 2018 ▸). It remains unknown whether the two active sites within a DHTKD1 dimer are engaged by one or two lipoyl domains. With either possibility, it is apparent that not all 24 lipoyl domains from one DLST core were engaged with DHTKD1 at the same time.

### 3.7. Structural mapping of disease-causing DHTKD1 mutations   

**Figure 4:**
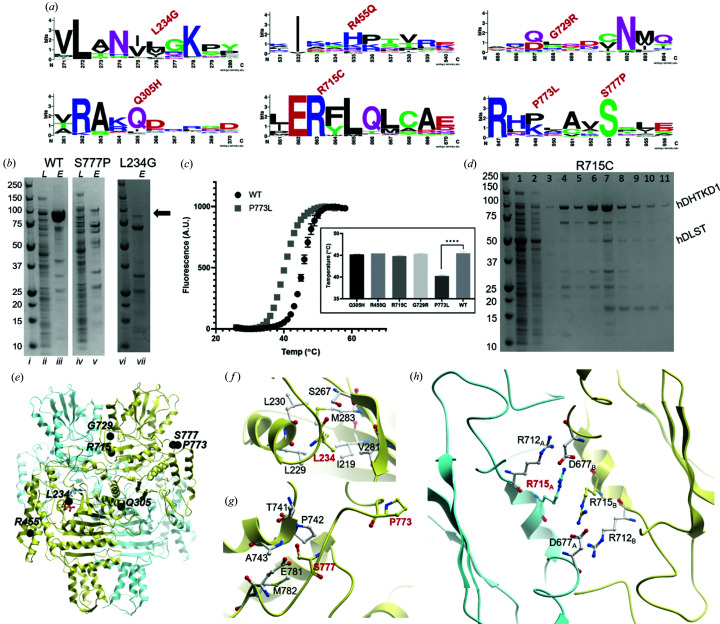
Characterization of hDHTKD1 disease-causing variants. (*a*) *Weblogo* diagrams generated from an alignment of >100 DHTKD1 orthologues and homologues, showing sequence conservation for the regions surrounding the seven missense mutation sites. (*b*) Small-scale expression and purification for hDHTKD1 WT and variants. SDS–PAGE gel slices showing lysate (*L*) and eluant (*E*) samples from affinity purification of hDHTKD1_45–919_ WT, p.S777P and p.L234G. The position of the hDHTKD1 bands is marked by an arrow. Original uncropped gels are shown in Fig. S13 (lanes *i*–*v* were cropped from one gel and lanes *vi*–*vii* were cropped from another). (*c*) DSF melting curves for hDHTKD1_45–919_ WT and p.P773L, with an inset showing the derived melting temperature (*T*_m_) values for WT and five variants. (*d*) Affinity pulldown of hDLST_68–453_ by immobilized His-tagged hDHTKD1_45–919_ p.R715C variant. The SDS–PAGE shown is one of two biological replicates (Fig. S15). The lanes are loaded with the following samples: 1, flow through; 2–6, wash fractions of increasing imidazole concentration; and 7–11, elution fractions with 250 m*M* imidazole. (*e*) hDHTKD1 homodimer showing the sites of the seven missense mutations (black spheres) on one subunit (yellow ribbon). (*f*)–(*h*) Atomic environments surrounding the mutation sites (*f*) p.L234G, (*g*) p.P773L and p.S777P, and (*h*) p.R715C. All amino acids shown, including sites of missense mutations, are of the WT protein.

To date, seven DHTKD1 missense mutations have been reported as the molecular cause for 2-amino­adipic and 2-oxoadipic aciduria. At the protein level, three (p.L234G, p.Q305H and p.R455Q) are located within the α/β1 domain, while the other four (p.R715C, p.G729R, p.P773L and p.S777P) are clustered in the α/β2 domain [Figs. 1 ▸(*a*) and 1 ▸(*c*)]. From over 100 DHTKD1 and OGDH orthologues surveyed, the aa 715 position is invariantly Arg, while aa positions 305 and 777 are also highly conserved (82% and 93%, respectively) [Fig. 4 ▸(*a*)]. None of these residues directly affect the conserved catalytic machinery common to the 2-oxoacid de­hydrogenase family.

To understand their putative biochemical defects, we reconstructed the seven DHTKD1 missense mutations recombinantly. All hDHTKD1_45–919_ variants were expressed as soluble protein to a similar level as WT, with the exception of p.L234G and p.S777P which showed significantly lower yields and propensity to degradation, suggesting these variant proteins are misfolded compared with WT [Figs. 4 ▸(*b*) and Fig. S13]. Leu234 is located at the protein centre ∼20 Å from the co-factor site [Fig. 4 ▸(*e*)] and the p.L234G change introduces a smaller side chain thereby leaving a cavity at the hydro­phobic core [Fig. 4 ▸(*f*)]. Ser777 is partially exposed to the protein surface [Fig. 4 ▸(*e*)] and the p.S777P change introduces a proline side chain that probably disrupts hydrogen bonds with neighbouring residues [Fig. 4 ▸(*g*)].

The five remaining variant proteins were isolated, purified and subjected to thermostability analysis by DSF. Four of them (p.Q305H, p.R455Q, p.R715C and p.G729R) demonstrated similar single unfolding–folding transition as hDHTKD1 WT [Fig. 4 ▸(*c*), inset]. However, p.P773L exhibited a significantly reduced melting temperature (Δ*T*_m_ = −5.2°C), suggesting that while expressed as soluble protein this variant is thermally more labile than WT [Fig. 4 ▸(*c*)]. Pro773 forms a bend for the surface-exposed loop which connects the α/β2 and α/β3 domains and also harbours the above-mentioned Ser777. Replacing Pro773 with Leu probably alters the structural integrity of this loop [Fig. 4 ▸(*h*)] and could affect protein folding. The observation of two destabilizing mutations within this one loop region strongly implies its importance in the functioning of DHTKD1.

Arg715 is located at the twofold axis of the homodimer and together with Arg712 forms a salt-bridge network with Asp677 of the opposite subunit [Fig. 4 ▸(*h*)]. Arg712, Arg715 and Asp677 are invariant amino acid positions across DHTKD1 and OGDH orthologues, indicating the importance of this salt-bridge network. Arg715 is positioned immediately after ‘loop 3’, a signature motif conserved across all E1 enzymes, including the invariant His708 that is involved in the reductive acyl transfer to E2 (Fig. S3) (Wynn *et al.*, 2003 ▸). SEC of the p.R715C variant revealed a similar chromatogram profile to WT hDHTKD1_45–919_, indicating intact dimer formation (Fig. S14). Nevertheless, when assayed in our co-expression and affinity pulldown, the hDHTKD1_45–919_ p.R715C variant has significantly reduced ability to bind hDLST_68–453_ directly [Figs. 4 ▸(*d*) and S15], compared with WT [Fig. 3 ▸(*b*)]. Therefore, the effect of p.R715C substitution could be transmitted from the dimer interface to engagement with E2 through loop 3. As a control, hDHTKD1_45–919_ bearing the p.R455Q or p.P773L substitution (both located at the protein exterior) interacts with hDLST_68–453_ to a similar extent as WT.

Our data did not reveal any discernible defects on protein stability or interaction with DLST *in vitro* for the p.Q305H, p.R455Q and p.G729R variants, the latter two being found in the majority of reported cases of 2-amino­adipic and 2-oxo­adipic aciduria. These results imply that additional functions or unknown binding partners could be involved in the OADHc. Future efforts can be focused on studying their *in vivo* impact using patient-relevant cells.

## 4. Conclusions   

DHTKD1 is emerging as a key player in mitochondrial metabolism through its influence in lysine metabolism, energy production and ROS balance. The structure of DHTKD1 presented here provides the first template for the rational design of DHTKD1 small-molecule modulators to probe the enzyme’s role in these mitochondrial functions and the associated disease states. DHTKD1 exhibits key structural differences from other E1 enzymes, particularly OGDH, with the key finding being that the active-site substrate pocket in DHTKD1 is larger in size and of more polar character than OGDH. These subtle modifications would favour the 2OA substrate, providing a molecular basis for the subtle difference in substrate specificity that was reported previously. The additional sequence and structural variations at the protein exterior would also impact on protein–protein interaction and explain how the two enzymes could, to some extent, engage with the cognate E2 and E3 components differently despite their close homology. These features are likely to be exploited by future chemistry efforts for the development of DHTKD1-specific modulators.

We have reconstituted the DHTKD1–DLST complex *in vitro* and demonstrated for the first time that complex formation is disrupted in some disease-causing variants, probably via indirect (*e.g.* destabilizing DHTKD1 to reduce its steady-state level) or direct (*e.g.* altering the binding interface of DHTKD1) mechanisms. These data underscore the importance of DHTKD1 functioning within the context of the OADHc complex. Our cryo-EM and complementary studies provide insight into how DLST forms a multimeric core to recruit multiple DHTKD1 protomers into this mega assembly. Considering that both DHTKD1 and OGDH recruit DLST as their E2 component, future studies are warranted to explore the existence of a ‘hybrid’ complex in which lipoyl domains from one DLST multimeric core could engage with both DHTKD1 and OGDH at the same time. This could allow crosstalk and regulation between the OADHc and OGDHc complexes for their concerted mitochondrial functions.

## 5. Related literature   

The following reference is cited in the supporting information for this article: Svergun *et al.* (1995 ▸).

## 6. Data availability   

X-ray coordinates and structure factors have been deposited to the Protein Data Bank (PDB) (accession code 6sy1). Cryo-EM data have been deposited to the Electron Microscopy Data Bank (EMDB) (accession code EMD-11014).

## Supplementary Material

Supporting table and figures. DOI: 10.1107/S205225252000696X/pw5012sup1.pdf

Supplementary File S1 - MIDAS protein-metabolite screening data. DOI: 10.1107/S205225252000696X/pw5012sup2.txt

PDB reference: DHTKD1, 6sy1

EMDB reference: EMD-11014

## Figures and Tables

**Table 1:** Summary of diffraction and refinement statistics The numbers in parentheses represent data in the highest-resolution shell.

Data collection	
Synchrotron radiation facility	Diamond Light Source
Beamline	I03
Detector	Dectris PILATUS3 6M
Wavelength (Å)	0.9762
Reflections (measured/unique)	145107 (13870)
Space group	*P*1
Unit-cell parameters	
*a, b, c* (Å)	78.55, 81.22, 86.90
α, β, γ (°)	63.43, 76.96, 72.06
Resolution (Å)	46.01–1.87 (1.937–1.87)
*R*_merge_	0.10 (0.44)
〈*I*/σ(*I*)〉	4.96 (1.04)
CC _1/2_	0.99 (0.64)
Completeness (%)	96.45 (91.94)
Multiplicity	1.8 (1.5)
Refinement	
Resolution (Å)	1.9
*R*_work_/*R*_free_ (%)	0.19/0.22
No. of reflections	
Working set	144961 (13819)
Test set	7088 (698)
Total no. of atoms	13331
Ligands at active site	55
No. of water molecules	1066
Wilson *B* factor (Å^2^)	21.81
RMS deviations	
Bond lengths (Å)	0.007
Bond angles (°)	0.84
Ramachandran plot	
Favoured (%)	96.9
Allowed (%)	2.87
Outliers (%)	0.23
Molecules in asymmetric unit	2
PDB code	6sy1

## References

[bb1] Adams, P. D., Afonine, P. V., Bunkóczi, G., Chen, V. B., Davis, I. W., Echols, N., Headd, J. J., Hung, L.-W., Kapral, G. J., Grosse-Kunstleve, R. W., McCoy, A. J., Moriarty, N. W., Oeffner, R., Read, R. J., Richardson, D. C., Richardson, J. S., Terwilliger, T. C. & Zwart, P. H. (2010). *Acta Cryst.* D**66**, 213–221. 10.1107/S0907444909052925 PMC2815670 20124702

[bb2] Ævarsson, A., Chuang, J. L., Max Wynn, R., Turley, S., Chuang, D. T. & Hol, W. G. (2000). *Structure*, **8**, 277–291. 10.1016/s0969-2126(00)00105-2 10745006

[bb67] Albert, A., Martinez-Ripoll, M., Espinosa-Ruiz, A., Yenush, L., Culianez-Macia, F. A., Serrano, R. (2000). *Structure*, **8**, 961 10.1016/s0969-2126(00)00187-8 10986463

[bb3] Araújo, W. L., Trofimova, L., Mkrtchyan, G., Steinhauser, D., Krall, L., Graf, A., Fernie, A. R. & Bunik, V. I. (2013). *Amino Acids*, **44**, 683–700. 10.1007/s00726-012-1392-x 22983303

[bb4] Bunik, V., Kaehne, T., Degtyarev, D., Shcherbakova, T. & Reiser, G. (2008). *FEBS J.* **275**, 4990–5006. 10.1111/j.1742-4658.2008.06632.x 18783430

[bb5] Bunik, V. I. & Brand, M. D. (2018). *Biol. Chem.* **399**, 407–420. 10.1515/hsz-2017-0284 29337692

[bb6] Bunik, V. I. & Degtyarev, D. (2008). *Proteins*, **71**, 874–890. 10.1002/prot.21766 18004749

[bb7] Bunik, V. I. & Sievers, C. (2002). *Eur. J. Biochem.* **269**, 5004–5015. 10.1046/j.1432-1033.2002.03204.x 12383259

[bb8] Burnley, T., Palmer, C. M. & Winn, M. (2017). *Acta Cryst.* D**73**, 469–477. 10.1107/S2059798317007859 PMC5458488 28580908

[bb10] Ciszak, E. M., Korotchkina, L. G., Dominiak, P. M., Sidhu, S. & Patel, M. S. (2003). *J. Biol. Chem.* **278**, 21240–21246. 10.1074/jbc.M300339200 12651851

[bb11] Cohen, R. D. & Pielak, G. J. (2017). *Protein Sci.* **26**, 403–413. 10.1002/pro.3092 PMC5326556 27977883

[bb12] Craig, D. W. & Wedding, R. T. (1980). *J. Biol. Chem.* **255**, 5763–5768. 6769921

[bb13] Danhauser, K., Sauer, S. W., Haack, T. B., Wieland, T., Staufner, C., Graf, E., Zschocke, J., Strom, T. M., Traub, T., Okun, J. G., Meitinger, T., Hoffmann, G. F., Prokisch, H. & Kölker, S. (2012). *Am. J. Hum. Genet.* **91**, 1082–1087. 10.1016/j.ajhg.2012.10.006 PMC3516599 23141293

[bb14] Duran, M., Beemer, F. A., Wadman, S. K., Wendel, U. & Janssen, B. (1984). *J. Inherit. Metab. Dis.* **7**, 61. 10.1007/BF01805803 6434826

[bb15] Emsley, P. & Cowtan, K. (2004). *Acta Cryst.* D**60**, 2126–2132. 10.1107/S0907444904019158 15572765

[bb16] Fischer, M. H., Gerritsen, T. & Opitz, J. M. (1974). *Humangenetik*, **24**, 265–270. 10.1007/BF00297590 4442872

[bb17] Frank, R. A., Price, A. J., Northrop, F. D., Perham, R. N. & Luisi, B. F. (2007). *J. Mol. Biol.* **368**, 639–651. 10.1016/j.jmb.2007.01.080 PMC7611002 17367808

[bb41] Franke, D., Petoukhov, M. V., Konarev, P. V., Panjkovich, A., Tuukkanen, A., Mertens, H. D. T., Kikhney, A. G., Hajizadeh, N. R., Franklin, J. M., Jeffries, C. M. & Svergun, D. I. (2017). *J. Appl. Cryst.* **50**, 1212–1225. 10.1107/S1600576717007786 PMC5541357 28808438

[bb18] Goncalves, R. L., Bunik, V. I. & Brand, M. D. (2016). *Free Radical Biol. Med.* **91**, 247–255. 10.1016/j.freeradbiomed.2015.12.020 26708453

[bb19] Hagen, J., te Brinke, H., Wanders, R. J., Knegt, A. C., Oussoren, E., Hoogeboom, A. J., Ruijter, G. J., Becker, D., Schwab, K. O., Franke, I., Duran, M., Waterham, H. R., Sass, J. O. & Houten, S. M. (2015). *J. Inherit. Metab. Dis.* **38**, 873–879. 10.1007/s10545-015-9841-9 25860818

[bb20] Holm, L. & Sander, C. (1995). *Trends Biochem. Sci.* **20**, 478–480. 10.1016/s0968-0004(00)89105-7 8578593

[bb21] Izard, T., Aevarsson, A., Allen, M. D., Westphal, A. H., Perham, R. N., de Kok, A. & Hol, W. G. (1999). *Proc. Natl Acad. Sci. USA*, **96**, 1240–1245. 10.1073/pnas.96.4.1240 PMC15447 9990008

[bb22] Jordan, F. (2003). *Nat. Prod. Rep.* **20**, 184–201. 10.1039/b111348h 12735696

[bb23] Jordan, F., Nemeria, N. & Gerfen, G. (2019). *Neurochem. Res.* **44**, 2325–2335. 10.1007/s11064-019-02765-w 30847859

[bb24] Kato, M., Wynn, R. M., Chuang, J. L., Brautigam, C. A., Custorio, M. & Chuang, D. T. (2006). *EMBO J.* **25**, 5983–5994. 10.1038/sj.emboj.7601444 PMC1698891 17124494

[bb25] Knapp, J. E., Mitchell, D. T., Yazdi, M. A., Ernst, S. R., Reed, L. J. & Hackert, M. L. (1998). *J. Mol. Biol.* **280**, 655–668. 10.1006/jmbi.1998.1924 9677295

[bb26] Krissinel, E. & Henrick, K. (2007). *J. Mol. Biol.* **372**, 774–797. 10.1016/j.jmb.2007.05.022 17681537

[bb27] Lawlis, V. B. & Roche, T. E. (1981). *Biochemistry*, **20**, 2519–2524. 10.1021/bi00512a024 6894547

[bb28] Leandro, J., Dodatko, T., Aten, J., Hendrickson, R. C., Sanchez, R., Yu, C., DeVita, R. J. & Houten, S. M. (2019). *bioRxiv*, https://doi.org/10.1101/2020.01.20.912931.

[bb29] Marrott, N. L., Marshall, J. J., Svergun, D. I., Crennell, S. J., Hough, D. W., van den Elsen, J. M. & Danson, M. J. (2014). *Biochem. J.* **463**, 405–412. 10.1042/BJ20140359 25088564

[bb30] Mattevi, A., Obmolova, G., Schulze, E., Kalk, K. H., Westphal, A. H., de Kok, A. & Hol, W. G. (1992). *Science*, **255**, 1544–1550. 10.1126/science.1549782 1549782

[bb31] McCartney, R. G., Rice, J. E., Sanderson, S. J., Bunik, V., Lindsay, H. & Lindsay, J. G. (1998). *J. Biol. Chem.* **273**, 24158–24164. 10.1074/jbc.273.37.24158 9727038

[bb32] McCoy, A. J., Grosse-Kunstleve, R. W., Storoni, L. C. & Read, R. J. (2005). *Acta Cryst.* D**61**, 458–464. 10.1107/S0907444905001617 15805601

[bb33] Nemeria, N. S., Gerfen, G., Nareddy, P. R., Yang, L., Zhang, X., Szostak, M. & Jordan, F. (2018). *Free Radical Biol. Med.* **115**, 136–145. 10.1016/j.freeradbiomed.2017.11.018 29191460

[bb34] Nemeria, N. S., Gerfen, G., Yang, L., Zhang, X. & Jordan, F. (2018). *Biochim. Biophys. Acta*, **1859**, 932–939. 10.1016/j.bbabio.2018.05.001 29752936

[bb35] Niesen, F. H., Berglund, H. & Vedadi, M. (2007). *Nat. Protoc.* **2**, 2212–2221. 10.1038/nprot.2007.321 17853878

[bb36] Orsak, T., Smith, T. L., Eckert, D., Lindsley, J. E., Borges, C. R. & Rutter, J. (2012). *Biochemistry*, **51**, 225–232. 10.1021/bi201313s 22122470

[bb37] Park, Y. H., Wei, W., Zhou, L., Nemeria, N. & Jordan, F. (2004). *Biochemistry*, **43**, 14037–14046. 10.1021/bi049027b 15518552

[bb38] Patel, M. S., Korotchkina, L. G. & Sidhu, S. (2009). *J. Mol. Catal. B Enzym.* **61**, 2–6. 10.1016/j.molcatb.2009.05.001 PMC2770179 20160912

[bb39] Perham, R. N. (1991). *Biochemistry*, **30**, 8501–8512. 10.1021/bi00099a001 1888719

[bb40] Perham, R. N., Jones, D. D., Chauhan, H. J. & Howard, M. J. (2002). *Biochem. Soc. Trans.* **30**, 47–51. 10.1042/12023822

[bb42] Pettersen, E. F., Goddard, T. D., Huang, C. C., Couch, G. S., Greenblatt, D. M., Meng, E. C. & Ferrin, T. E. (2004). *J. Comput. Chem.* **25**, 1605–1612. 10.1002/jcc.20084 15264254

[bb43] Quinlan, C. L., Goncalves, R. L., Hey-Mogensen, M., Yadava, N., Bunik, V. I. & Brand, M. D. (2014). *J. Biol. Chem.* **289**, 8312–8325. 10.1074/jbc.M113.545301 PMC3961658 24515115

[bb44] Reed, L. J. (2001). *J. Biol. Chem.* **276**, 38329–38336. 10.1074/jbc.R100026200 11477096

[bb45] Reed, L. J. & Hackert, M. L. (1990). *J. Biol. Chem.* **265**, 8971–8974. 2188967

[bb46] Rigden, D. J. & Galperin, M. Y. (2004). *J. Mol. Biol.* **343**, 971–984. 10.1016/j.jmb.2004.08.077 15476814

[bb47] Rohou, A. & Grigorieff, N. (2015). *J. Struct. Biol.* **192**, 216–221. 10.1016/j.jsb.2015.08.008 PMC6760662 26278980

[bb48] Rutter, G. A., McCormack, J. G., Midgley, P. J. & Denton, R. M. (1989). *Ann. NY Acad. Sci.* **573**, 206–217. 10.1111/j.1749-6632.1989.tb14998.x 2699397

[bb49] Sauer, S. W., Okun, J. G., Schwab, M. A., Crnic, L. R., Hoffmann, G. F., Goodman, S. I., Koeller, D. M. & Kölker, S. (2005). *J. Biol. Chem.* **280**, 21830–21836. 10.1074/jbc.M502845200 15840571

[bb50] Scheres, S. H. & Chen, S. (2012). *Nat. Methods*, **9**, 853–854. 10.1038/nmeth.2115 PMC4912033 22842542

[bb51] Sherrill, J. D., Kc, K., Wang, X., Wen, T., Chamberlin, A., Stucke, E. M., Collins, M. H., Abonia, J. P., Peng, Y., Wu, Q., Putnam, P. E., Dexheimer, P. J., Aronow, B. J., Kottyan, L. C., Kaufman, K. M., Harley, J. B., Huang, T. & Rothenberg, M. E. (2018). *JCI Insight*, **3**, e99922. 10.1172/jci.insight.99922 PMC5931135 29669943

[bb52] Stein, N. (2008). *J. Appl. Cryst.* **41**, 641–643.

[bb53] Svergun, D., Barberato, C. & Koch, M. H. J. (1995). *J. Appl. Cryst.* **28**, 768–773.

[bb54] Svergun, D. I. (1992). *J. Appl. Cryst.* **25**, 495–503.

[bb55] VanderJagt, D. J., Garry, P. J. & Hunt, W. C. (1986). *Clin. Chem.* **32**, 1004–1006. 3708799

[bb56] Wagner, T., Barilone, N., Alzari, P. M. & Bellinzoni, M. (2014). *Biochem. J.* **457**, 425–434. 10.1042/BJ20131142 24171907

[bb57] Wagner, T., Bellinzoni, M., Wehenkel, A., O’Hare, H. M. & Alzari, P. M. (2011). *Chem. Biol.* **18**, 1011–1020. 10.1016/j.chembiol.2011.06.004 21867916

[bb58] Wagner, T., Boyko, A., Alzari, P. M., Bunik, V. I. & Bellinzoni, M. (2019). *J. Struct. Biol.* **208**, 182–190. 10.1016/j.jsb.2019.08.012 31476368

[bb9] Winn, M. D., Ballard, C. C., Cowtan, K. D., Dodson, E. J., Emsley, P., Evans, P. R., Keegan, R. M., Krissinel, E. B., Leslie, A. G. W., McCoy, A., McNicholas, S. J., Murshudov, G. N., Pannu, N. S., Potterton, E. A., Powell, H. R., Read, R. J., Vagin, A. & Wilson, K. S. (2011). *Acta Cryst.* D**67**, 235–242. 10.1107/S0907444910045749 PMC3069738 21460441

[bb59] Wynn, R. M., Machius, M., Chuang, J. L., Li, J., Tomchick, D. R. & Chuang, D. T. (2003). *J. Biol. Chem.* **278**, 43402–43410. 10.1074/jbc.M306204200 12902323

[bb60] Xu, W., Zhu, H., Gu, M., Luo, Q., Ding, J., Yao, Y., Chen, F. & Wang, Z. (2013). *FEBS Lett.* **587**, 3587–3592. 10.1016/j.febslet.2013.08.047 24076469

[bb61] Xu, W. Y., Gu, M. M., Sun, L. H., Guo, W. T., Zhu, H. B., Ma, J. F., Yuan, W. T., Kuang, Y., Ji, B. J., Wu, X. L., Chen, Y., Zhang, H. X., Sun, F. T., Huang, W., Huang, L., Chen, S. D. & Wang, Z. G. (2012). *Am. J. Hum. Genet.* **91**, 1088–1094. 10.1016/j.ajhg.2012.09.018 PMC3516600 23141294

[bb62] Yeaman, S. J. (1989). *Biochem. J.* **257**, 625–632. 10.1042/bj2570625 PMC1135633 2649080

[bb63] Yu, X., Hiromasa, Y., Tsen, H., Stoops, J. K., Roche, T. E. & Zhou, Z. H. (2008). *Structure*, **16**, 104–114. 10.1016/j.str.2007.10.024 PMC4807695 18184588

[bb64] Zheng, S. Q., Palovcak, E., Armache, J. P., Verba, K. A., Cheng, Y. & Agard, D. A. (2017). *Nat. Methods*, **14**, 331–332. 10.1038/nmeth.4193 PMC5494038 28250466

[bb65] Zhou, J., Yang, L., Ozohanics, O., Zhang, X., Wang, J., Ambrus, A., Arjunan, P., Brukh, R., Nemeria, N. S., Furey, W. & Jordan, F. (2018). *J. Biol. Chem.* **293**, 19213–19227. 10.1074/jbc.RA118.005432 PMC6302161 30323066

[bb66] Zhou, Z. H., McCarthy, D. B., O’Connor, C. M., Reed, L. J. & Stoops, J. K. (2001). *Proc. Natl Acad. Sci. USA*, **98**, 14802–14807. 10.1073/pnas.011597698 PMC64939 11752427

