# Supplementary material for: Crystal structure and interaction studies of human DHTKD1 provide insight into a mitochondrial megacomplex in lysine catabolism
Source: IUCrJ. 2020 Jun 10;7(Pt 4):693–706. doi: 10.1107/S205225252000696X (PMC7340257; doi:10.1107/S205225252000696X)
Supplement: Supplementary file 1 [file m-07-00693-sup1.pdf]

IUCrJ

Volume 7 (2020)

Supporting information for article:

Crystal structure and interaction studies of human DHTKD1 provide insight into a mitochondrial megacomplex in lysine catabolism

Gustavo A. Bezerra, William R. Foster, Henry J. Bailey, Kevin G. Hicks, Sven W. Sauer, Bianca Dimitrov, Thomas J. McCorvie, Juergen G. Okun, Jared Rutter, Stefan Koelker and Wyatt W. Yue

Table S1 Cryo-EM data collection and 3D reconstruction

	**DLST PDB EMD-11014**
**Data Collection**	
Microscope	Glacios
Voltage (keV)	200
Nominal Magnification	150,000 x
Total electron exposure (e/Å^2)	32.52
Camera	Falcon3
Fractions	19
Exposure (sec)	1
Pixel size (Å)	0.96
Nominal defocus range (μm)	-1 to -3.1
Micrographs collected (no.)	619
Final refined particles (no.)	3356
**Reconstruction**	
Symmetry imposed	O
Resolution (Å) (global) FSC	4.7
Applied B-factor (Å^2)	-281
**Model refinement statistics**	
Refmac5 FSC average (%)	73
Rms angle	1.41
Rms bond	0.0056
Rms chiral	0.0858
Model vs Map at FSC 0.5 (Å)	6.5
Model vs Map FSC average >	81

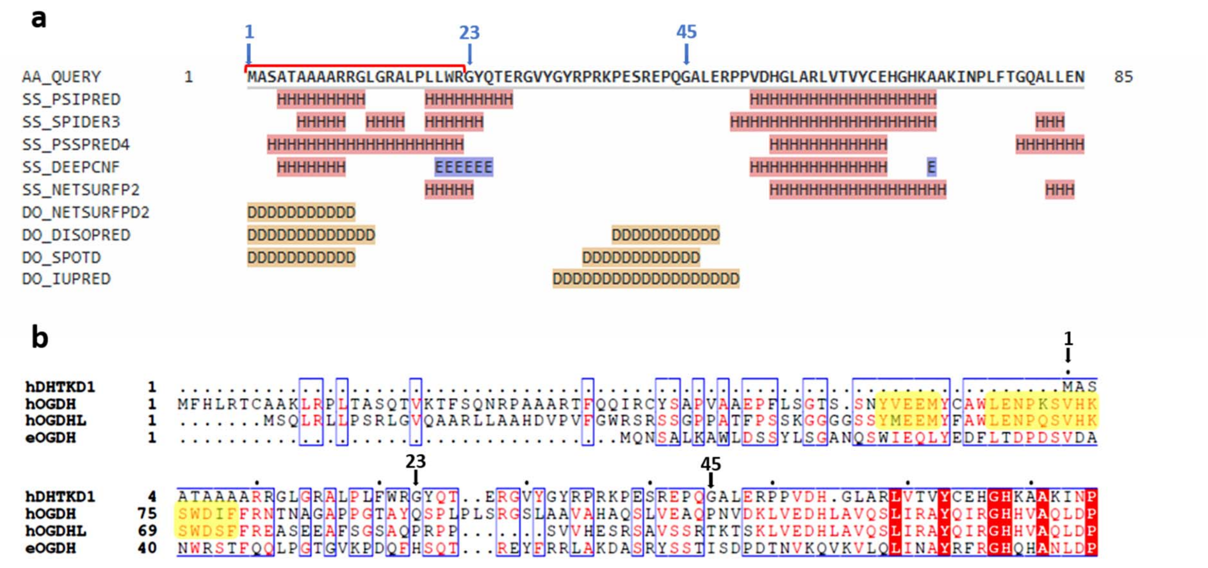

Figure S1 The N-terminus of human DHTKD1. (**a**) Secondary structure and intrinsic disorder prediction for the human DHTKD1 N-terminal 88 aa, from 9 different servers (H, helices; E, strand, D, disordered). The putative mitochondrial target sequence is bracketed (red). We have expressed DHTKD1 constructs that begin at residues 1, 23 or 45 (arrows). (**b**) Sequence-based alignment of the N-terminus from human DHTKD1, OGDH, OGDHL and E. coli OGDH. Motifs from OGDH (also conserved in OGDHL) that were mapped to interact with DLST (Orsak *et al.*, 2012) are shaded yellow.

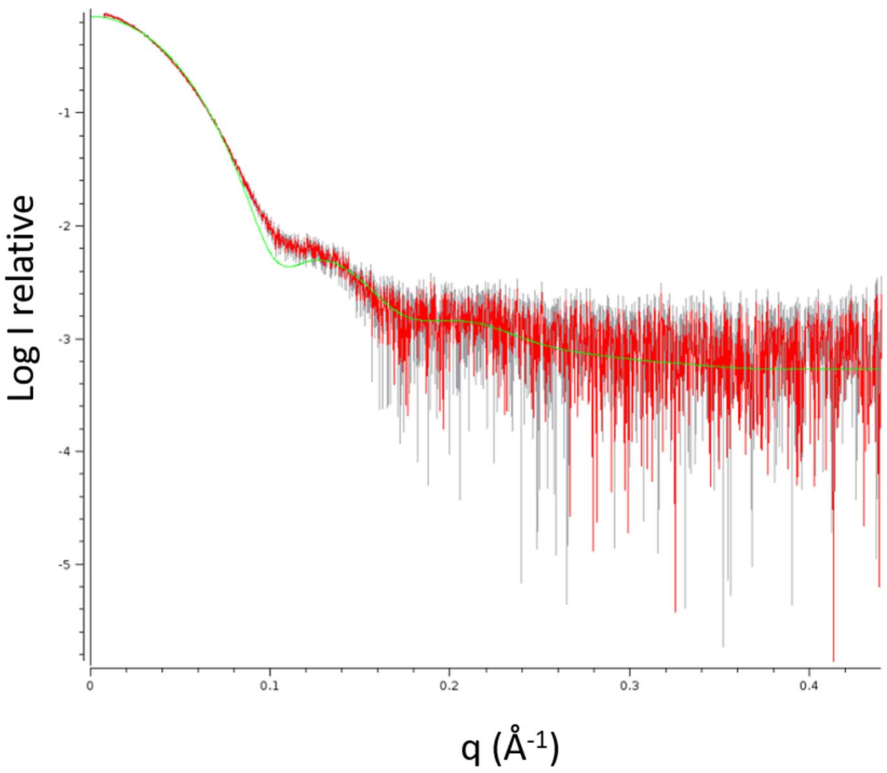

Figure S2 **SAXS analysis of hDHTKD1$_{45\text{-}919}$.** Overlay of experimental scattering profile (red) and theoretical scattering curve of DHTKD1 crystal dimer (green). A Chi2 fit of 3.8 was determined by CRYSOL (Svergun *et al.*, 1995).

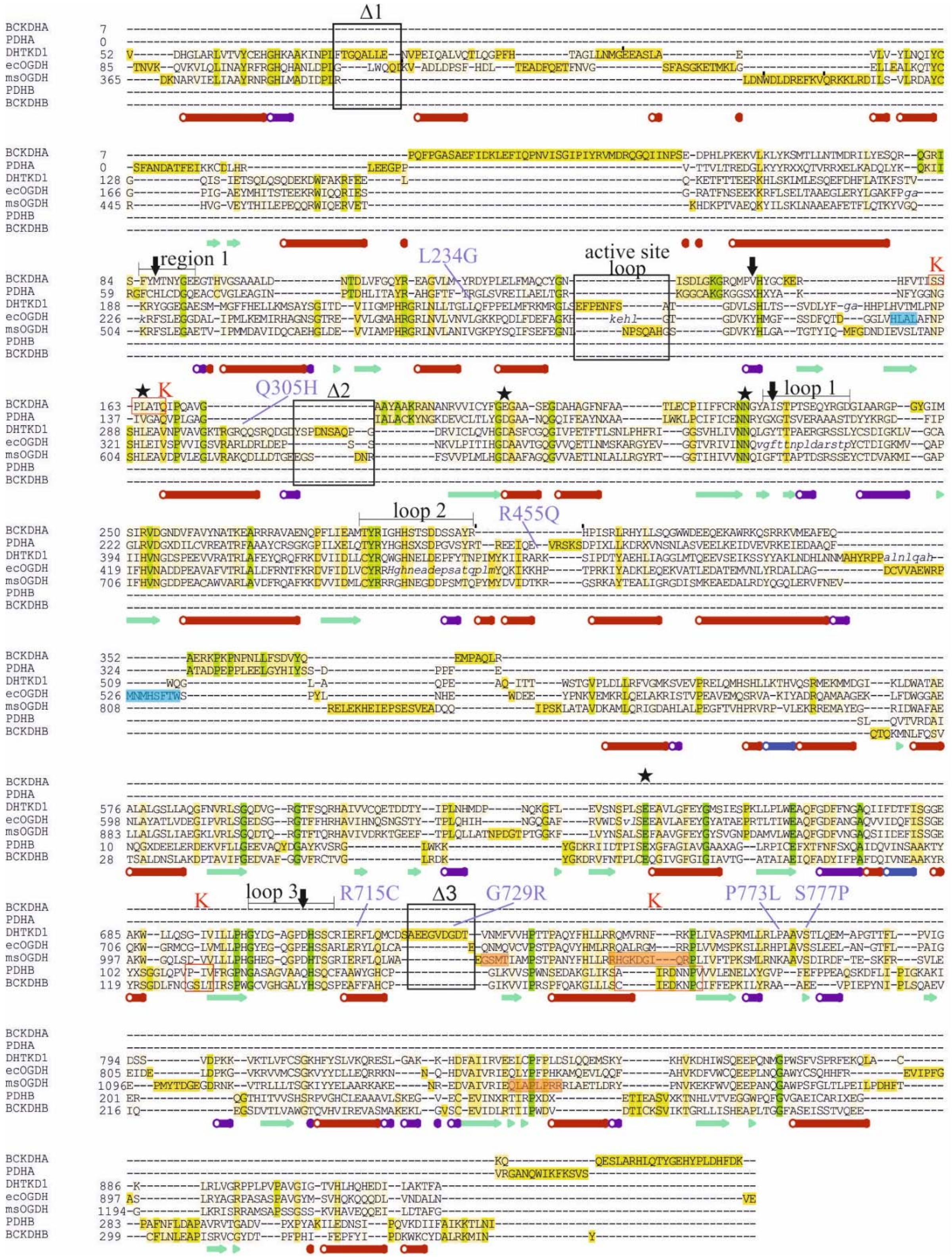

Figure S3 **Structure-based sequence alignment of representative 2-oxoacid dehydrogenases.** Aligned structures using *ICM Pro* (www.molsoft.com) include human DHTKD1 (this study), human human branched-chain α-ketoacid dehydrogenase subunits BCKDHA, BCKDHB (PDB 1dtw_a, 1dtw_b), human pyruvate dehydrogenase subunits PDHA, PDHB (1ni4_a, 1ni4_b), *E. coli* 2-

oxoglutarate dehydrogenase ecOGDH (2jgd), *M. Smegmatis* α-ketoglutarate decarboxylase msOGDH (2y0p). Residues that were not modelled in crystal structures are shown in italicized lowercase. Secondary structures for DHTKD1 are shown at the bottom. Annotated features shown on the alignment include: sites of missense mutations for DHTKD1 (purple fonts), DHTKD1-unique loop regions (black boxes), 4 amino acid positions at the DHTKD1 active site that are distinct from OGDH (black arrows), catalytic residues (black stars) and loop regions (black brackets) conserved across the 2-oxoacid dehydrogenase family, allosteric AMP (letters in blue shade) and acetyl-CoA (letters in orange shade) binding sites from ecOGDH and msOGDH structures respectively, and K^+ ion binding sites from human PDH and BCKDH structures (red boxes and font).

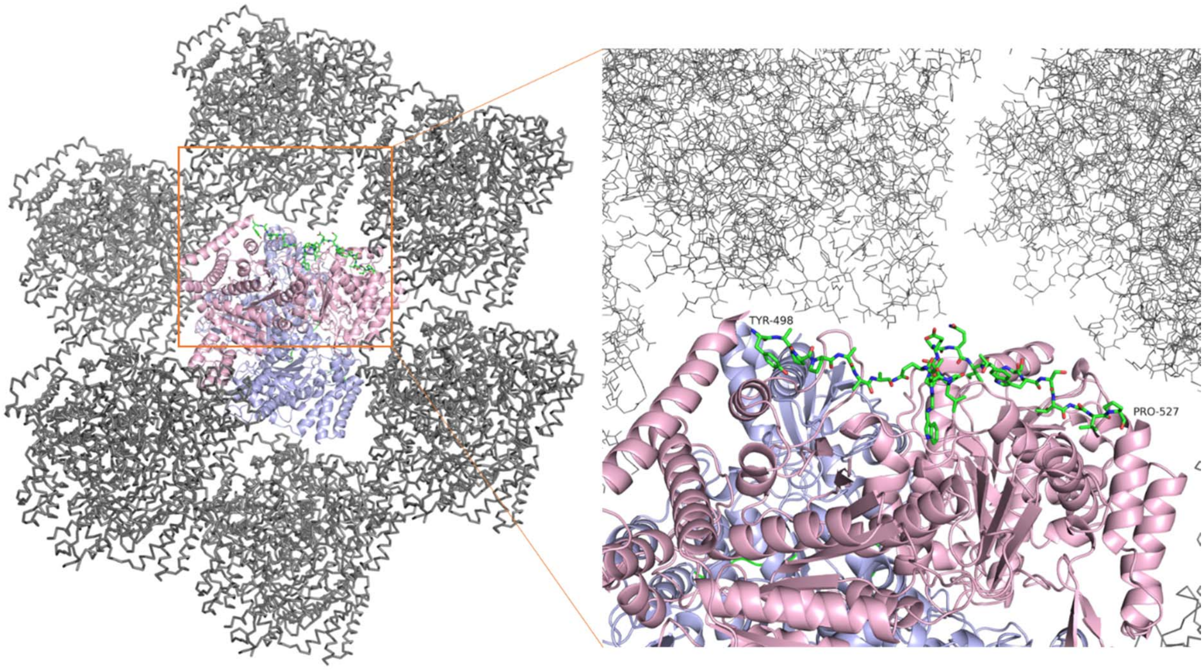

Figure S4 DHTKD1 crystal packing organisation. Overall arrangement of DHTKD1 symmetry mates shown as ribbons. Inset: Close-up view of the inter-domain linker (green sticks) from one subunit, with respective to the symmetry mates. The DHTKD1 homodimer from one asymmetric unit is depicted as pink and blue cartoons, with one inter-domain linker as green sticks. The symmetry mates are depicted as grey ribbons or grey lines.

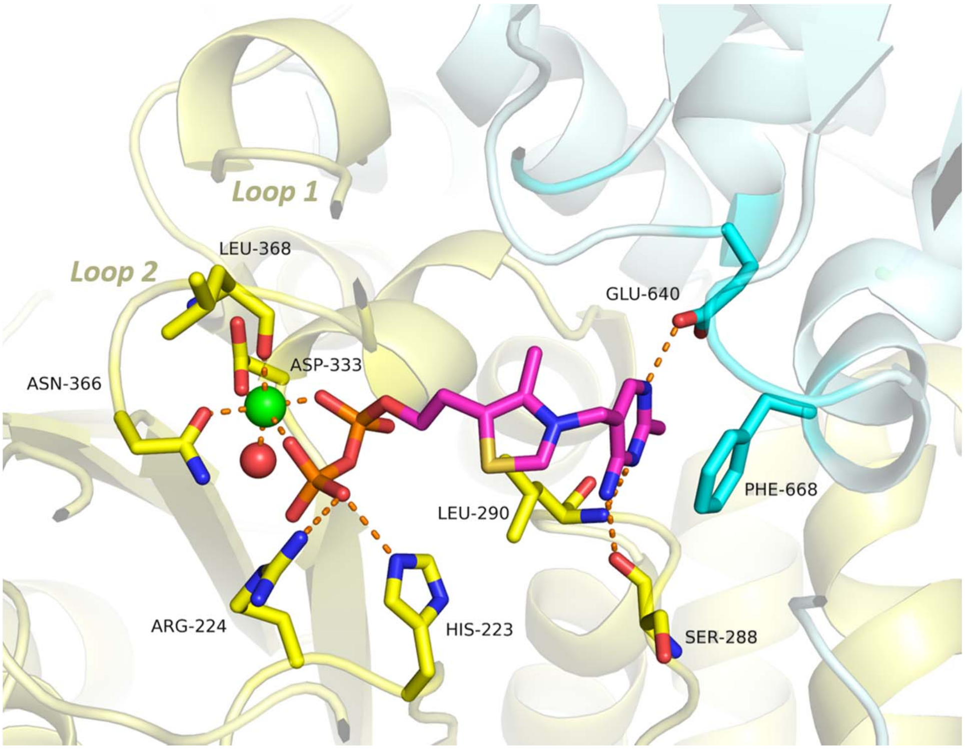

Figure S5 Binding site of the ThDP cofactor. ThDP (sticks, magenta carbon) binds to a pocket formed by residues from both subunits of the DHTKD1 homodimer (yellow and cyan ribbon/sticks). Mg^{2+} ion is shown as green sphere and a water molecule as red sphere. Hydrogen bonds are depicted as orange dashed lines.

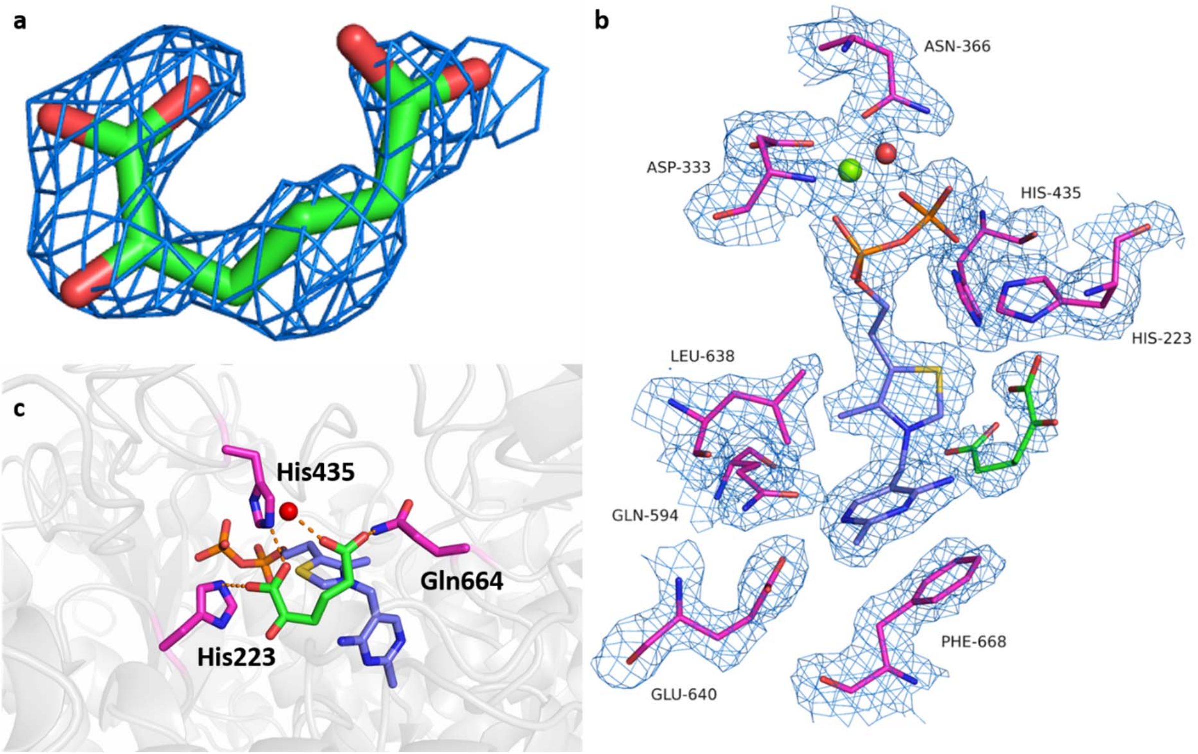

Figure S6 Putative 2-oxoadipate at the active site. (**a**) Simulated-annealing 2Fo-Fc composite omit map of hDHTKD1 contoured at 1 σ and displaying the density at the active site, into which a molecule of 2-oxoadipate was modelled. (**b**) Simulated-annealing 2Fo-Fc composite omit map of hDHTKD1 contoured at 1 σ and displaying ThDP as blue lines and the interacting residues as lilac lines. (**c**) Binding environment of the modelled ligand, showing that one carboxyl group of the ligand forms hydrogen bonds with Gln664 and a water molecule, while the other carboxyl group forms hydrogen bonds with His435 and His223. Shown in stick representation are: the modelled ligand with green carbon atoms, ThDP cofactor with lilac carbon atoms, and protein residues with magenta carbon atoms. Water molecules are presented as red spheres and Mg^{2+} as green sphere.

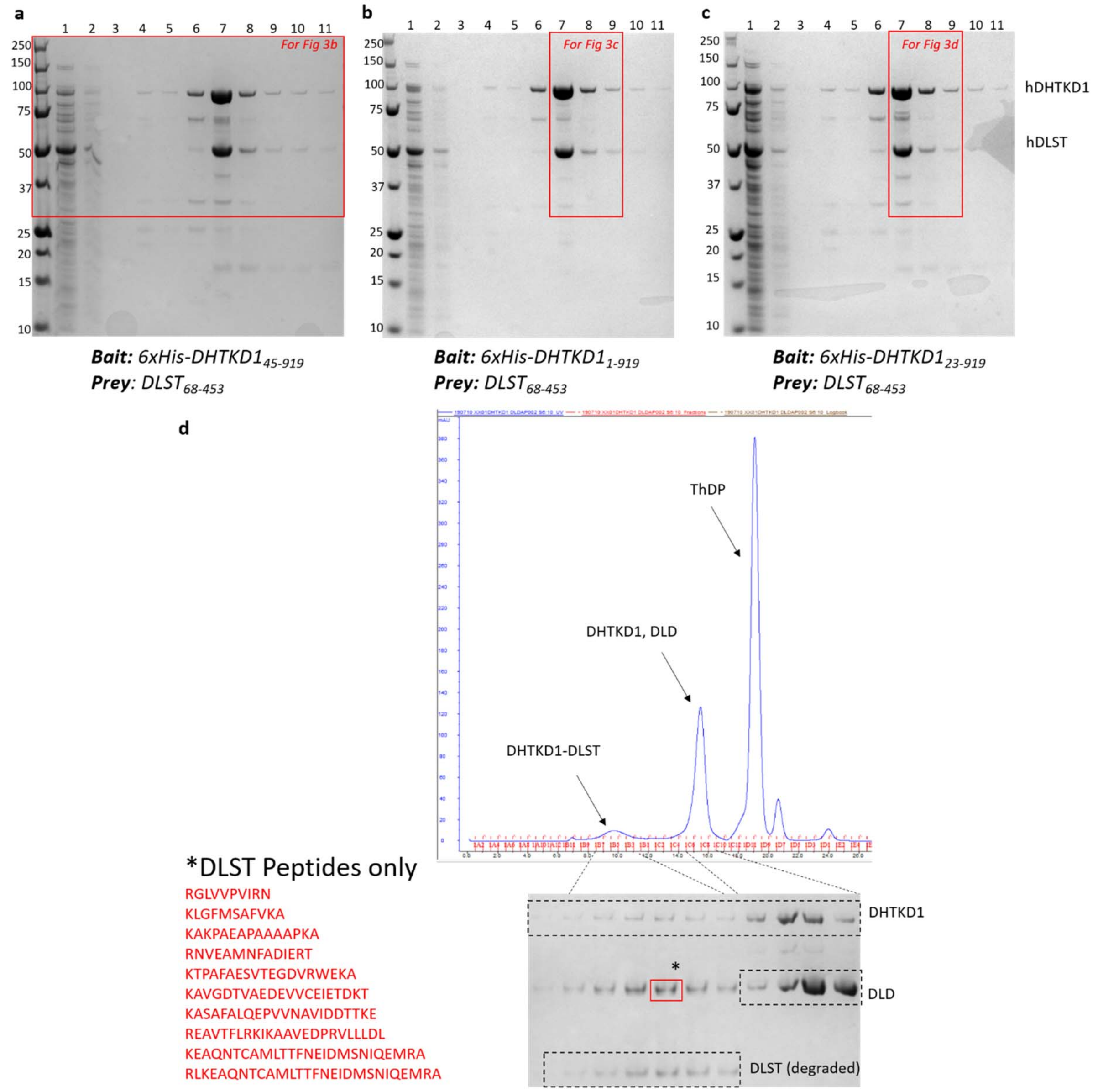

Figure S7 Affinity pull-down of DLST by immobilised His-tagged DHTKD1. Recombinant untagged $hDLST_{68\text{-}453}$ co-eluted with His6-tagged $hDHTKD1_{45\text{-}919}$ (**a**), $hDHTKD1_{1\text{-}919}$ (**b**), and $hDHTKD1_{23\text{-}919}$ (**c**). Full SDS-PAGE gels are shown here. Areas of the gels excised for display in Fig. 3b, c, and d of the main text are boxed. Lanes are loaded with following samples from Ni-affinity chromatography: 1, flow-through; 2-6, wash fractions of increasing imidazole concentration; 7-11, elution fractions with 250 mM imidazole. (**d**) Size exclusion chromatography (SEC) profile of pre-formed DHTKD1-DLST complex mixed with DLD. Bottom, SDS-PAGE analysis of SEC elution fractions shows that DLD did not elute in fractions containing DHTKD1-DLST, but instead appeared in a later elution volume. Peptides identified from tryptic digest MSMS analysis of one band (red box) is shown.

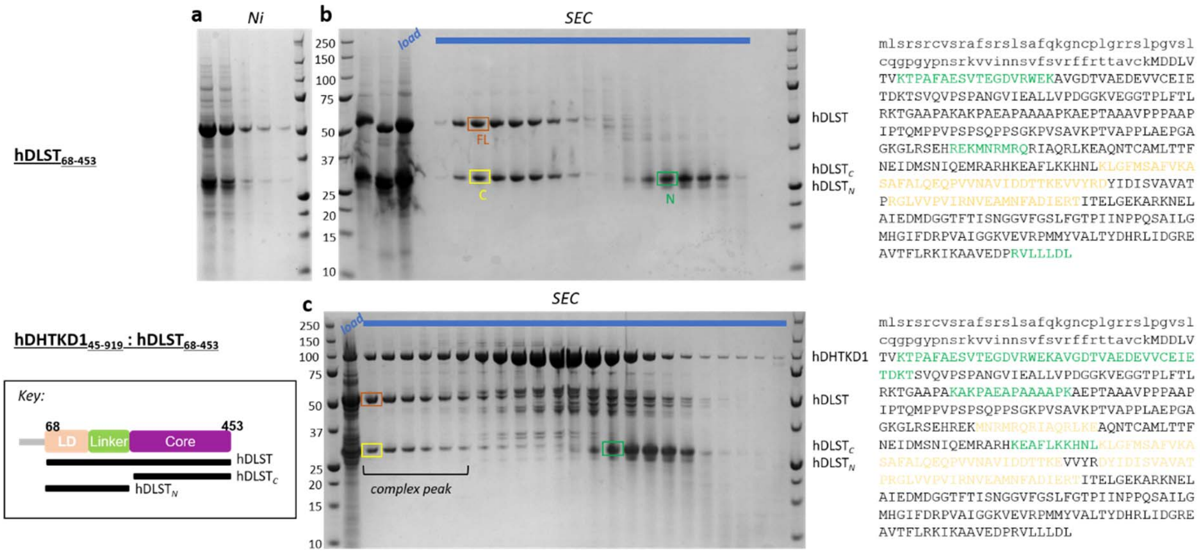

Figure S8 Purification of human DLST expressed from baculo Sf9 cells. (**a**) Elution fractions from Ni affinity purification of hDLST$_{68\text{-}453}$. (**b**) Elution fractions (blue bar) from size exclusion chromatography (HiLoad 16/600 Superdex 200) of the hDLST$_{68\text{-}453}$ sample partially purified from Ni affinity chromatography (as in panel A). Bands in boxes labelled FL, N, C have been verified by tryptic-MS/MS to contain full-length hDLST$_{48\text{-}653}$, and the N- and C-terminal halves. Peptides identified from the bands labelled N and C are coloured green and yellow on the human DLST sequence to the right (aa 1-67 not present in construct, in lowercase; aa 68-453 in uppercase). (**c**) Elution fractions (blue bar) from size exclusion chromatography (XK 16/70 Superose 6 prep grade) of hDLST$_{68\text{-}453}$ co-expressed with hDHTKD1$_{45\text{-}919}$ in insect Sf9 cells. Peak fractions from the DHTKD1-containing complex contain full-length as well as the C-terminal half of DLST, but not its N-terminal half. As with panel b, peptides revealed from tryptic-MS/MS analysis are shown to the right of the panel.

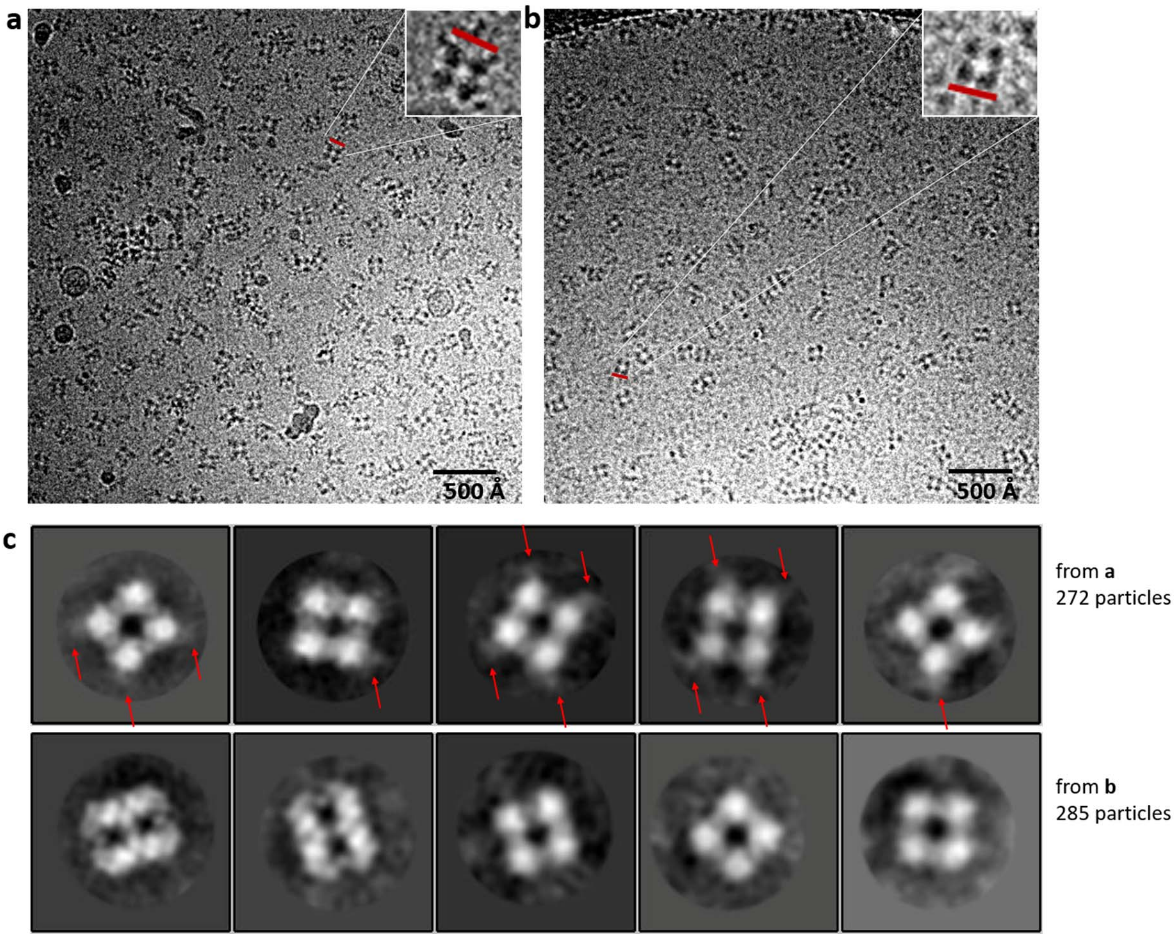

Figure S9 Electron Micrographs and 2D classifications representing two different samples of the DHTKD1-DLST complex. (**a**) The *E. coli* co-expressed complex reveals heterogenous particles with additional density located at the corners of the DLST cubes. (**b**) The Sf9 co-expressed complex reveals homogenous density of the DLST core catalytic domain only. Red bars indicate a length of approximately 130 Å. (**c**) Five 2D classes of the *E. coli* co-expressed complex compared with the Sf9 co-expressed complex. Red arrows indicate areas with additional heterogenous density.

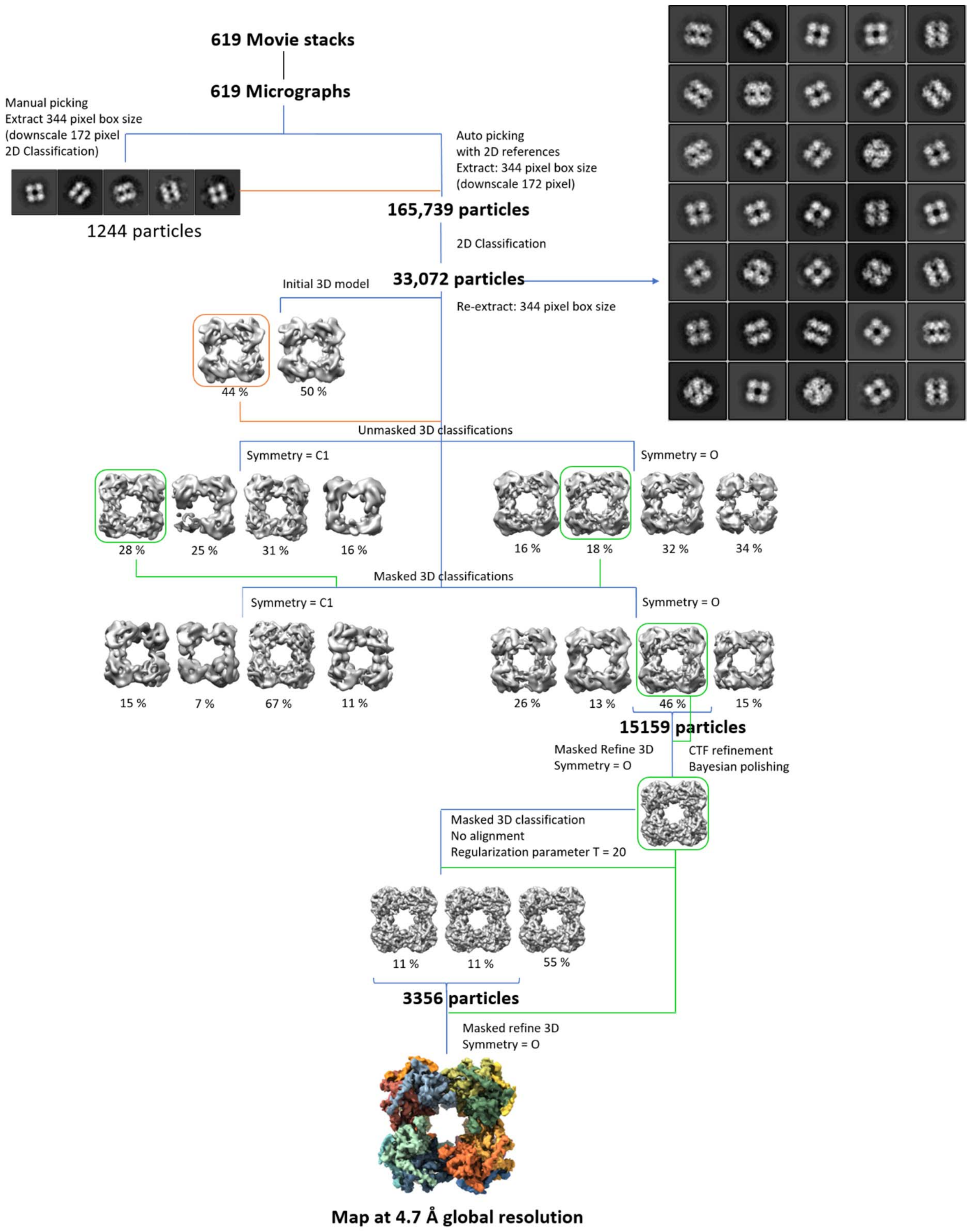

Figure S10 Schematic of the processing workflow that resulted in the final 3D reconstruction of the human DLST cube at 4.7 Å. Blue lines represent particles filtered through classification and refinement steps, Orange lines/boxes represent classes used as references in subsequent steps and green lines/boxes represent classes used as both references and mask generation for subsequent steps.

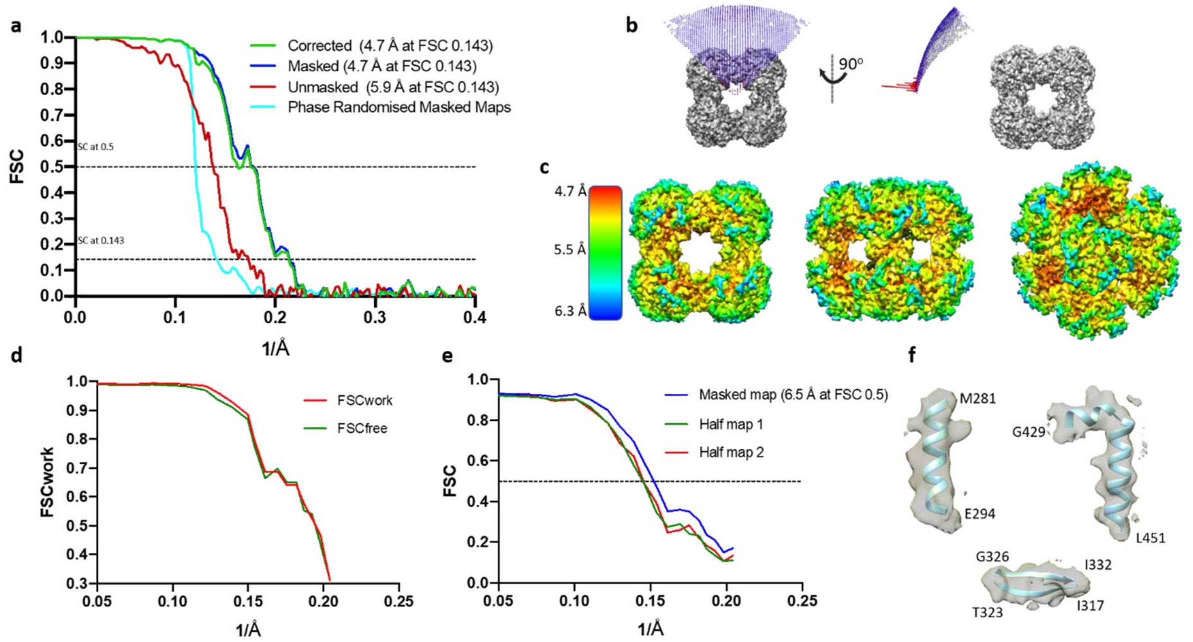

Figure S11 EM data validation and maps. (**a**) Gold standard FSC curve of phase randomised map showing global resolution of 4.7 Å for the masked map and 5.9 Å without masking. (**b**) Orientation distribution of the 3356 particles used in the final map reconstruction. (**c**) Local resolution of the maps scaled between 4.7 and 6.3 Å. (**d**) Overfitting was monitored through simultaneous refinement validation against half map 1 and half map 2 from the 3D reconstruction. (**e**) Map vs Model FSC calculated to be 6.5 Å when FSC = 0.5 and average FSC at 81 % for FSC >0.5. (**f**) Representative density and secondary structure fit to map.

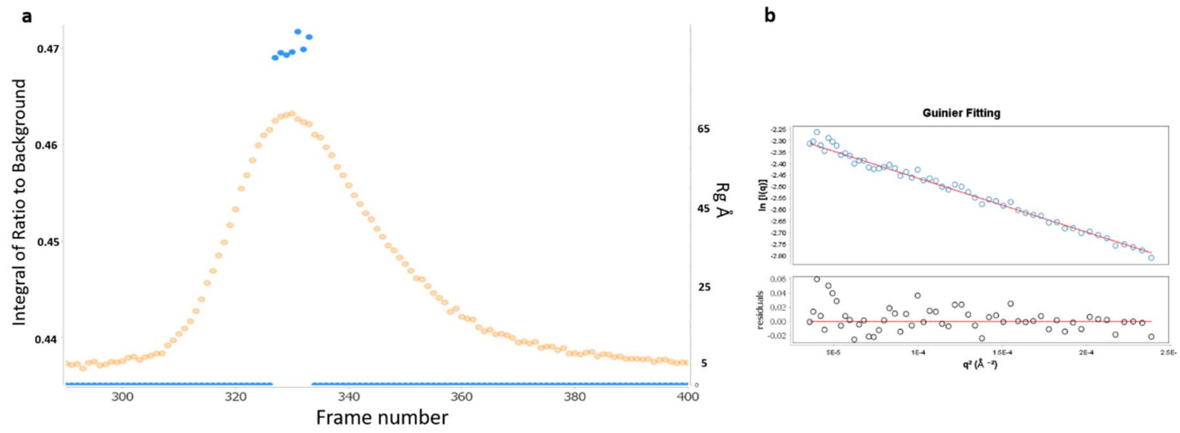

Figure S12 SEC-SAXS analysis of DHTKD1-DLST complex. (**a**) Analysis of the radius of gyration (Rg) over elution peak. The Rg (blue spheres) and Integral of Ratio to Background (yellow spheres) are plotted against recorded frames in SEC-SAXS profiles for the hDHTKD1$_{45\text{-}919}$:hDLST$_{68\text{-}453}$ complex co-expressed in Sf9 cells. The frames used for further analysis are 328-332. Exposure of 3 seconds per frame was recorded. (**b**) Rg value derived from the Guinier plot is 83.2 Å.

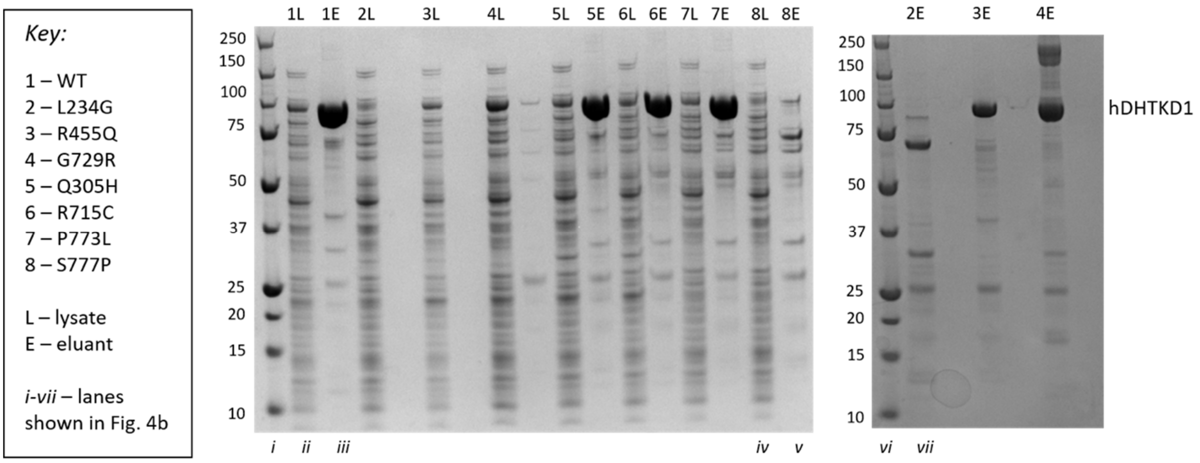

Figure S13 Small scale expression and purification test for DHTKD1 wt and variants. SDS-PAGE of lysate (L) and eluant (E) fractions from affinity purification of hDHTKD1$_{45\text{-}919}$ wt (1) and missense variants (2-8) are shown. Lanes excised for display in Fig. 4b are labelled in roman numerals i-vii.

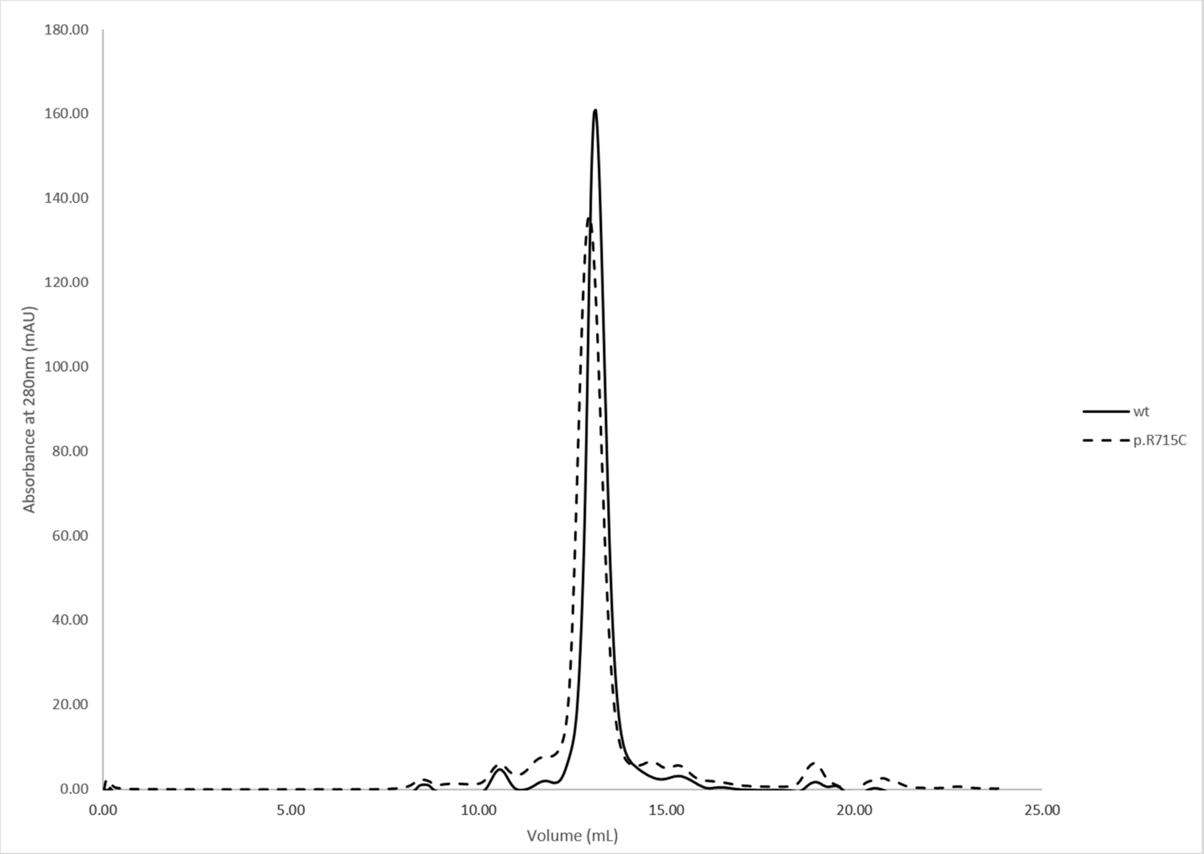

Figure S14 Size exclusion chromatography of DHTKD1 wt and p.R715C variant. Chromatograms from Superdex 200 Increase 10/300 GL column are overlayed, showing that both wt and variant proteins behave similarly in SEC, eluting at the same volume.

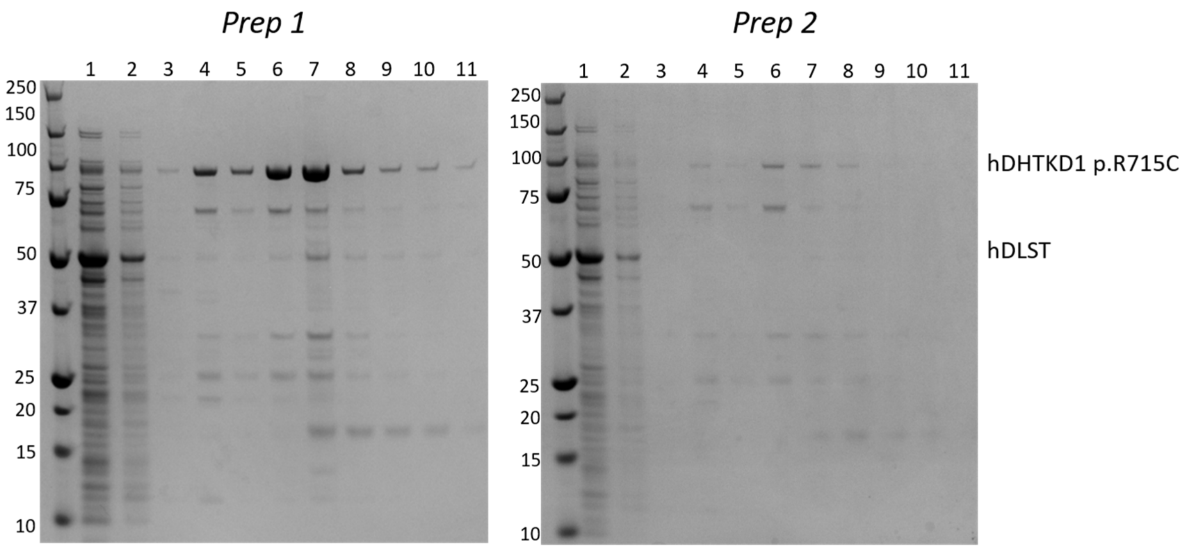

Figure S15 Affinity pulldown of DLST by immobilised His-tagged DHTKD1 p.R715C variant. SDS-PAGE are shown from two experimental replicates of different biological preparations. Lanes are loaded with following samples from Ni affinity chromatography: 1, flow-through; 2-6, wash fractions of increasing imidazole concentration; 7-11, elution fractions with 250 mM imidazole. SDS-PAGE for prep 1 is also shown in Fig. 4d.
